# High precision zero-friction magnetic dendrometer

**DOI:** 10.1016/j.ohx.2021.e00248

**Published:** 2021-11-12

**Authors:** Cameron Clonch, Mark Huynh, Bryson Goto, Alexander Levin, John Selker, Chet Udell

**Affiliations:** a Openly Published Environmental Sensing (OPEnS) Lab, Oregon State University, Corvallis, OR, USA; b Department of Horticulture, Oregon State University, Corvallis, OR, USA; c Southern Oregon Research and Extension Center, Oregon State University, Central Point, OR, USA; d Department of Biological & Ecological Engineering, Oregon State University, Corvallis, OR, USA

**Keywords:** Vineyard, Woody plants, Arduino, Carbon fiber, Irrigation, Water-stress indicator, Maximum daily shrinkage (MDS), Trunk growth rate

## Abstract

Increasing agricultural demand for freshwater in the face of a changing climate requires improved irrigation management to maximize resource efficiency. Soil water deficits can significantly reduce plant growth and development, directly impacting crop quantity and quality. Dendrometers are a plant-based tool that have shown potential to improve irrigation management in high-value woody perennial crops (e.g., trees and vines). A dendrometer continuously measures small fluctuations in stem diameter; this has been directly correlated to water stress measurements using traditional methods. While plant-based measures of water deficits are considered to be the best measures of water stress, current dendrometer methods are imprecise due to mechanical hysteresis and thermal expansion. The high-precision dendrometer created at the OPEnS Lab alleviates these key failure points using zero-thermal expansion carbon fiber, zero friction via a spring tensioning approach, and a linear magnetic encoder. In-lab tests and field deployments have validated device measurements and the execution of these pivotal qualities. Mass deployment of these automated dendrometers has the potential to provide a continuous record of water stress, providing valuable decision support for irrigation management.

## Graphical abstract

**Figure:**
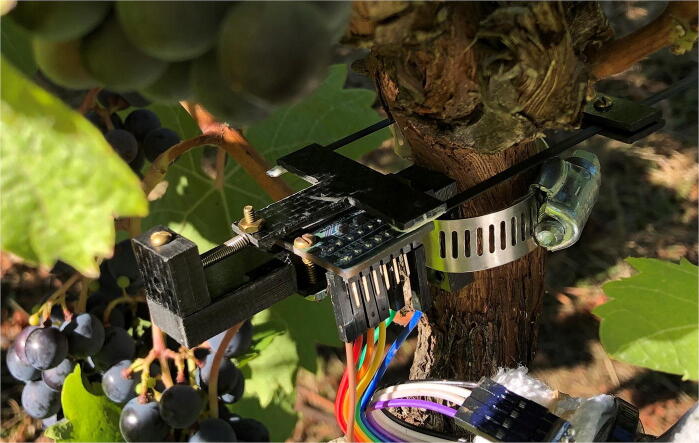

**Specifications table:** 

Hardware name	*Zero-friction Dendrometer*
Subject area	Environmental, Planetary and Agricultural Sciences
Hardware type	Field measurements and sensors
Open Source License	GNU GPL v3, CERN OHL-S v2
Cost of Hardware	*$260 (US) per Dendrometer*
Source File Repository	GitHub: https://github.com/OPEnSLab-OSU/Dendrometer; Zenodo: https://doi.org/10.5281/zenodo.4482948

## 1 Hardware in context

Understanding crop water stress provides valuable information regarding plant development that can assist in optimizing irrigation scheduling. Water scarcity in regions common for fruit production, coupled with an increasing world population, makes the use of precise irrigation techniques in vineyards and orchards essential [1]. Continuous measurements of plant water stress can optimize water application by informing irrigators on precisely when irrigation is needed. Ultimately, this leads to improved productivity and water conservation, sustaining essential agro- and natural ecosystems. This increased robustness can have long-lasting effects on the overall socio-economic well-being of agricultural areas.

Plant water deficits correlate to altered, sometimes more desirable, fruit physiology and development. For example, in grapevines (*Vitis vinifera L.*), targeted water stress can change sugar content, fruit skin characteristics, color, wine aroma, berry size, and more. The timing of this water stress can impact grape and wine quality [2], [3]. Indeed, growers of red wine grapes often purposefully employ water deficits to increase accumulation of certain metabolites and alter the ratio of berry skin to flesh [4]. Active evaluation of plant water stress provides means for enhanced crop customization and better understanding plant physiology.

Traditionally, irrigation management decision support has relied on measurements of soil water content (SWC) or other soil-based measurements. Measuring SWC is straightforward (e.g., Meter Group Teros 12 [5]), simple to automate and collect continuous measurements, and data interpretation is well-documented. However, the major disadvantage of soil-based measurements is that they sample a small soil volume that is not representative of the soil volume explored by plant roots, especially under drip irrigation [6]. This disadvantage is magnified in perennial crops with a large rooting volume such as trees and vines. Soil-based sensors often imprecisely represent plant water availability. One solution is to use the plant itself as the sensor of soil water availability with plant-based methods.

Current methods for the acquisition of highly accurate plant water stress data are expensive, discontinuous, and/or inaccessible for users without advanced training. The most widely used and accepted tool is the pressure chamber that measures leaf or stem water potential [7]. To use the pressure chamber, a leaf sample must be bagged, excised from the plant, placed into a sealed chamber with the cut end of the leaf petiole protruding through an airtight grommet, and pressurized with inert compressed gas. When sap appears at the cut end, pressurization is stopped, and the value on the chamber gauge is recorded – the higher the value, the more severe the water deficit. However, samples can only be collected during a narrow window in time (2–3 h), and to date there is no way to automate the procedure. This necessitates that large producers with disparate fields employ multiple operators, further compounding potential measurement errors [8]. Thus, a significant input of labor is necessary to properly monitor plant water status and widespread integration of the required equipment is not feasible; this method cannot realistically provide continuous, real-time measurements [9].

Another plant-based option is to measure sap flow using sensors embedded in the trunks of trees or vines. These tools allow for continuous and automated data collection, but variations in this sap flow do not pinpoint water stress as the source [1]. Measurements from identical probes in the same plant can vary significantly due to nuances in probe location and positioning [10], as well as species and individual variances in how sap flows in different parts of each plant. Finally, setup, maintenance, and data interpretation require a high level of training that makes them inaccessible to most producers.

Ultimately, the nature of soil-based measurements and existing plant-based measurements greatly limit how often measurements can be taken and the feasibility of acquiring a multitude of data. Holistic, real-time evaluations of crops are nearly unachievable and resources for such operations are simply unavailable. The dendrometer created by the OPEnS Lab can facilitate broad evaluation of woody perennial crops, as well as collect plant specific data, fulfilling various scales of crop evaluation.

Dendrometers quantify plant stress by continuously measuring stem diameter. Plant stem diameter oscillates diurnally through periods of expansion and contraction. These oscillations correlate inversely with stress associated with plant transpiration; e.g., when the plant is well-watered, oscillations will be less pronounced (lower amplitude), whereas when experiencing a water deficit and transpiration is reduced, the oscillations will be more extreme (higher amplitude) [6]. Currently available dendrometers rely on potentiometers or linear variable placement transducers for evaluating stem diameter changes [9]. Some of the most common mechanical designs include the band dendrometer, such as the Model 9605 BEI by Duncan Electronics [11], and the point dendrometer, like the Natkon Dendrometer ZN12-O-WP [12]. These devices tend to lack precision and accuracy due to the limitations of the technology employed. In particular, band and point dendrometers encounter a significant amount of friction that leads to inaccurate measurements. Delays from “stickiness” due to seals between sliding parts lead to hysteresis: as the stem switches between expanding and contracting, the instrument is unable to measure and record these micrometer-level motions. Since the amplitude of these variations is central to the method, this defect is problematic. Finally, a potentiometer restricts the form factor of the mechanical design and exhibits strong temperature dependency, sacrificing the ability to achieve high-resolution measurements. As outdoor air temperature changes, the dendrometer materials/components will experience expansion/contraction as well, often impossible to distinguish from the true changes in the stem dimensions.

The dendrometer produced at the OPEnS lab seeks to solve these problems. By using a frictionless mechanism, mechanical hysteresis is eliminated, facilitating accurate quantification of fluctuations in diameter. Further, the use of a magnetic encoder, as opposed to a potentiometer, enables high-precision measurements to 0.5 μm. Moreover, the OPEnS Dendrometer is constructed of carbon fiber, a material known for its extremely low coefficient of thermal expansion [13]. This ensures that material expansion/contraction does not confound with measuring the diameter of the plant stem.

A more accurate, precise, and accessible dendrometer can help advance agricultural water management so that it is more responsive to the crop’s immediate needs. The refinement of such technology is crucial for advancing agriculture towards a more sustainable future.

## 2 Hardware description

**Fig. 1:**
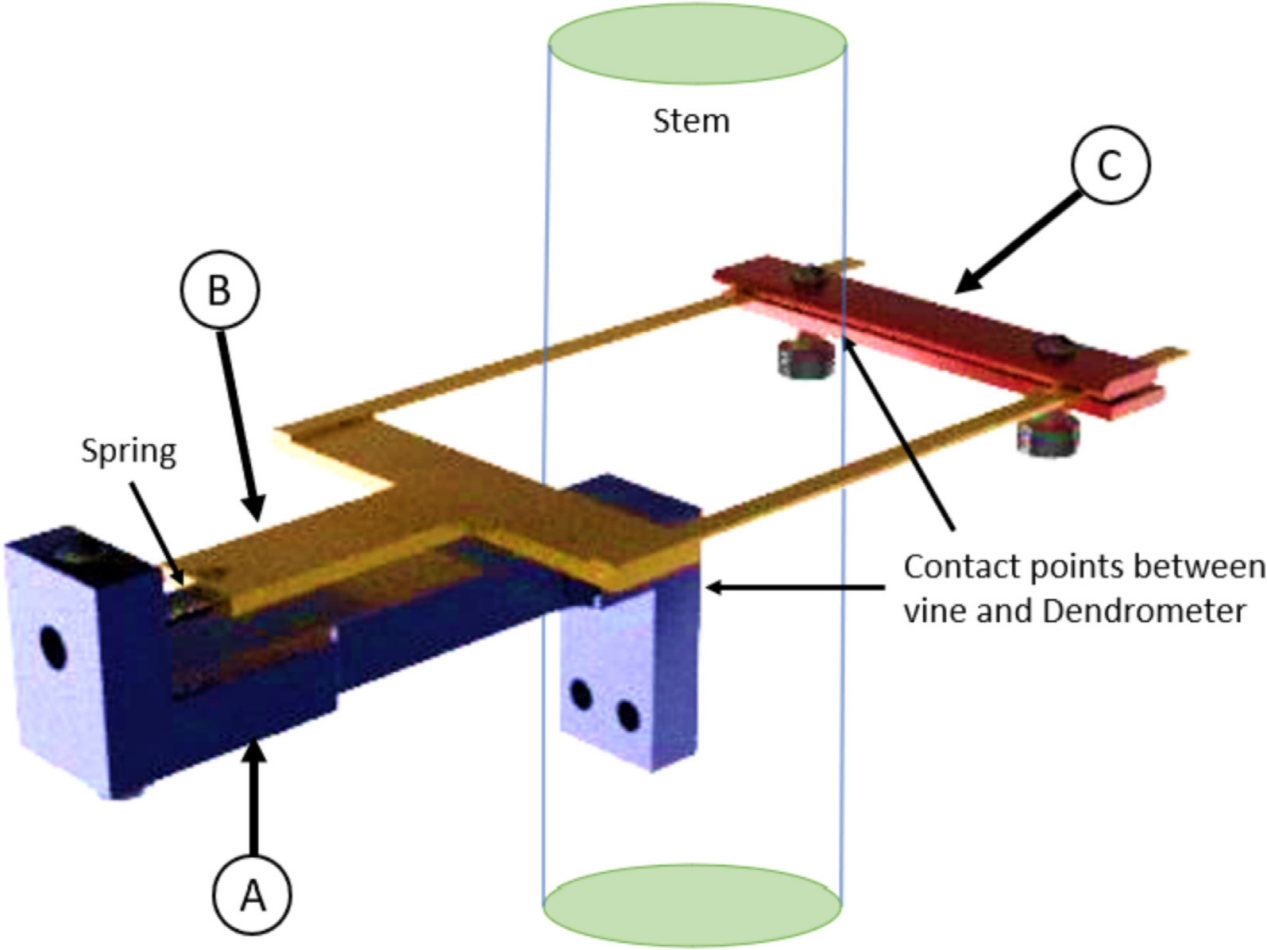
3D Model of OPEnS Dendrometer mechanical components: A) frame (blue elements); B) T Magnet Mount (yellow elements); C) Sandwich Grip (red elements). All mechanical components, except fasteners and the spring, are made from carbon fiber. (For interpretation of the references to color in this figure legend, the reader is referred to the web version of this article.)

The dendrometer can be broken down into three main functional components: 1) frame (Fig. 1: A), which includes the Long Body, Vine Contact Mount, and Spring Hold; 2) magnet holder, composed of the T Magnet Mount (Fig. 1: B), rods, and Sandwich Grip (Fig. 1: C); and 3) electronics. Stem diameter expansions and contractions move the magnet holder assembly relative to the stationary frame and attached AS5311 magnetic sensor [14].

### 2.1 Frame

The frame is held against the nearside of the vine using a hose clamp. Wood screws can also be used.

### 2.2 Magnet holder

The magnet holder secures the magnet relative to the vine and the spring by utilizing a T-shaped cutout on the nearside of the vine (Fig. 1: B, magnet is secured with adhesive on the underside) joined via rods to an adjustable sandwiching piece (aka the Sandwich Grip) on the far side (Fig. 1: C). The edge of the Sandwich Grip (Fig. 1: C) is installed against the farside of the vine so that it lightly pulls on the spring. The spring provides tension to keep the Sandwich Grip in contact with the stem and also enables the magnet holder assembly to move linearly relative to the frame (Fig. 1: A). The Sandwich Grip (Fig. 1: C) at the end of the rods is removable and placement is adjustable to fit stems 25 mm – 40 mm in diameter. However, the dendrometer design and dimensions can be modified to accommodate other stems sizes by widening the distance between the rods and lengthening the rods.

### 2.3 Electronics

**Fig. 2:**
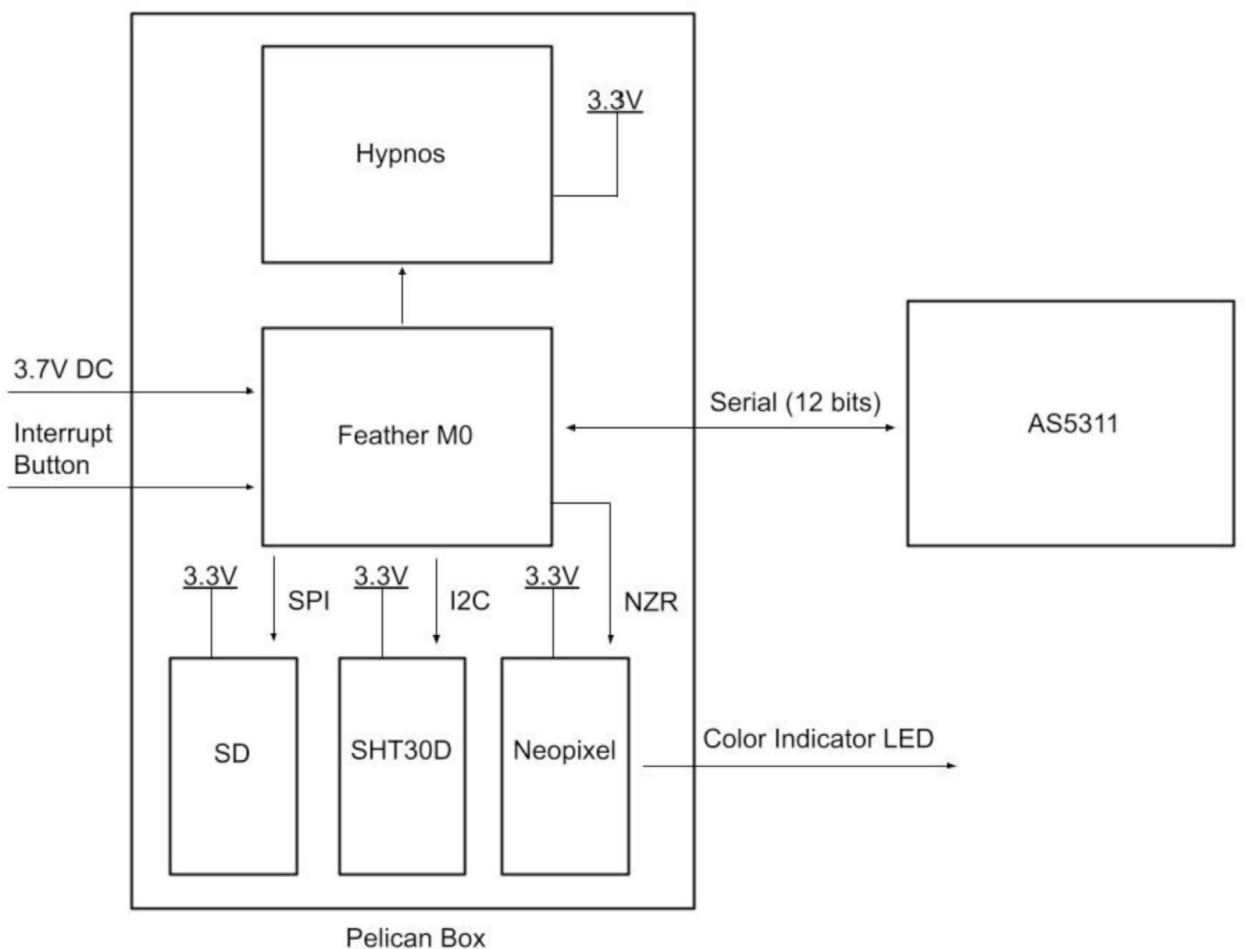
Block diagram of dendrometer electronics. System-level overview of components.

The electronics include the AS5311 linear magnetic sensor, custom printed circuit board (PCB) for capturing stem diameter fluctuations at a resolution of 0.5 µm, Hypnos PCB [15] for powering the sensors, real-time clock, and data logging functions, as well as the Feather M0 microprocessor (Fig. 2). The 3.7 V LiPo battery is connected to the Feather which is then connected to the Hypnos board for 3.3 V power switching. The sensors are then connected to the Hypnos for the 3.3 V supply.

### 2.4 Innovations

Carbon fiber is lightweight and has a near-zero coefficient of thermal expansion [13]. Materials employed by other dendrometers include Invar and stainless steel with coefficients of thermal expansion of 1.5 µm/(m°C) and 16 µm/(m°C) respectively [16]. To quantify the consequences for Invar and stainless steel, a 10 °C temperature fluctuation over 24 h (i.e. a low of 15 °C and high of 25 °C) can induce material expansion that leads to 15 to 210 µm/m, thus typically about 2–20 µm change in dimensions for a 10-cm instrument. Expansion and contraction of the device materials can skew data and limit reliability and accuracy. When the material expands, the instrument measures fluctuations in both the tool and the plant as opposed to only the plant stem. Given the small magnitude of diurnal fluctuations in vines (typically 100–150 µm [9]), minimizing temperature sensitivity is imperative for accurate crop evaluation and providing useful guidance on crop care.

**Fig. 3:**
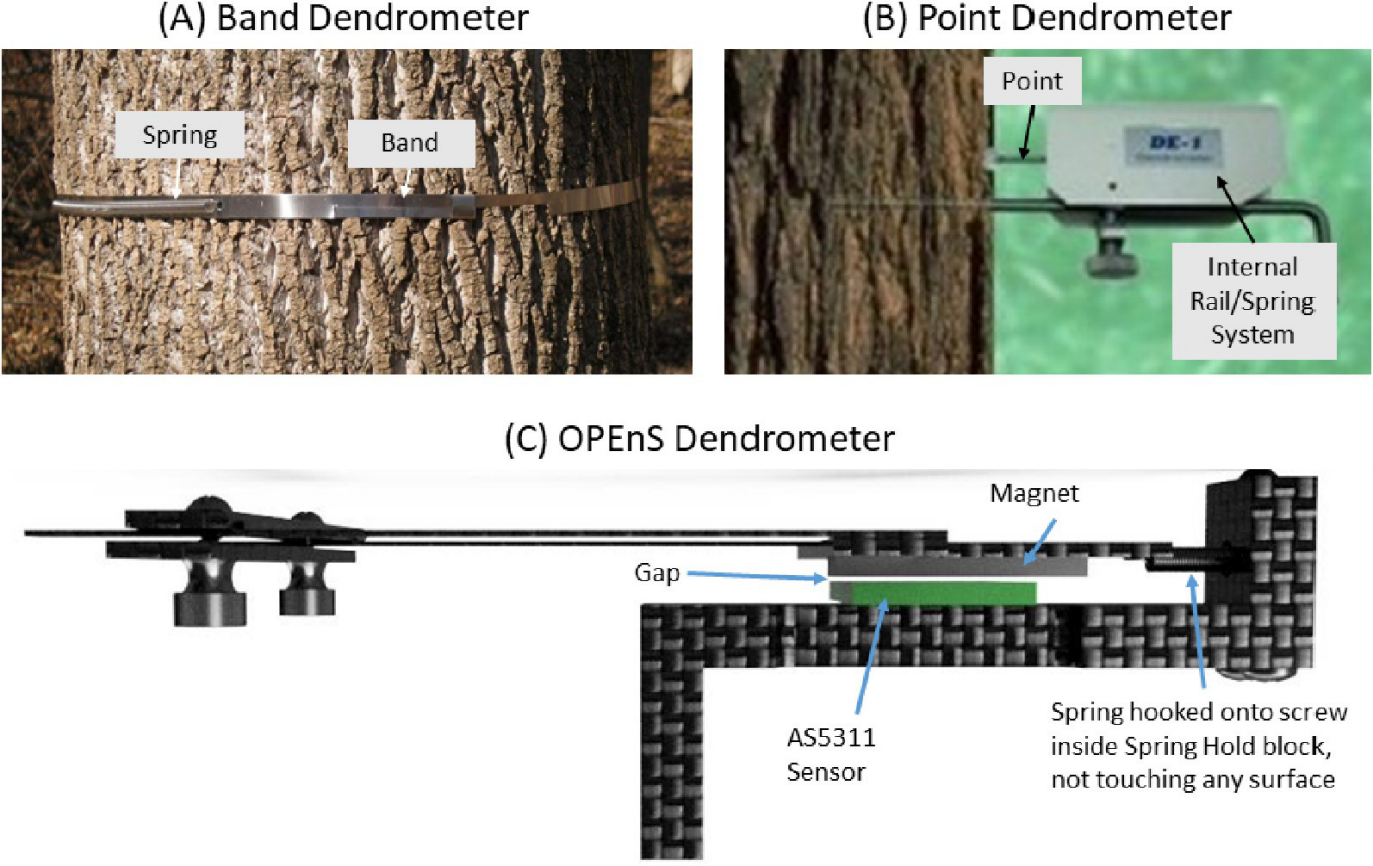
Comparison between band (A), point (B), and OPEnS (C) dendrometers. Point and band dendrometers contain elements of rubbing or sliding that can create inaccuracies or hysteresis when dealing with micrometer fluctuations. A band dendrometer has a spring and band mechanism that can rub against each other, as well as on the bark [17]. The point dendrometer relies on a rail-style system to guide the needle point that pushes into the tree [18]. Both the band and point dendrometer design are subject to hysteresis, leaving micrometer fluctuations in stem diameter unrecorded.

Another essential advantage of the OPEnS Dendrometer is the frictionless mechanical system. By relying on a spring-loaded design, all movement recorded by the magnetic sensor is guided by tension; there is no mechanical contact between parts in opposition that generally impedes upon measurement accuracy. This design mechanism is not found in any other dendrometer available on the market. The OPEnS dendrometer allows the spring to move freely; there is no rubbing on any device surface and the magnet floats above the sensor (Fig. 3: C). The magnet and spring will respond to changes in the single contact point between the Sandwich Grip and the vine on the far side of the stem.

Using a linear magnetic sensor (AS5311), the OPEnS Dendrometer tracks diurnal changes in vine diameter. The AS5311 can accurately (0.5 μm resolution) decode the linear displacement of a multi-pole (alternating north and south) magnetic strip that is hovering above the sensor. To achieve stated accuracy for the AS5311 sensor, the pole pairs of the magnet must be 2.0 mm long and the magnet must also have a resolution of 10 µm/m or better. The position of these pole pairs directly maps to absolute values for the sensor, which are deciphered via firmware to allow the dendrometer to track position over time. The firmware that is written for the OPEnS Dendrometer communicates serially with the Synchronous Serial Interface (SSI) and uses on-board memory to calculate and preserve the overall linear displacement of the magnet over time.

The OPEnS Dendrometer includes an LED indication system that enables the user to easily verify that the device is properly installed. It is essential that the magnet and AS5311 sensor maintain a certain separation distance; the LED indication system is used to check that this requirement is met. Upon initial installation, the LED automatically signals alignment status. The system can also be used to check the alignment during periods of data collection by pushing a button, which will illuminate the LED; the color of the light reflects device status. This improves device usability because the SD card does not have to be removed to determine if usable data is being collected.

The device employs an SHT30 Temperature and Humidity sensor, which is used to record ambient temperature and humidity (these values can be used to calculate vapor-pressure deficit). This sensor includes a weatherproof casing and other adjustments to make it easily deployable in the field. It uses I2C to communicate with the microprocessor and measures environmental temperatures from −40 °C to 125 °C as well as humidity from 20%RH to 80%RH [19].

The open-sourced nature of the project reduces consumer costs significantly. Currently available dendrometers cost around $1000 (US) each [12], whereas the OPEnS Dendrometer, built without economies of scale, is approximately $260 (US). This opens the possibility of mass deployment throughout a field or vineyard, providing stronger, more reliable indications of crop health and water stress. Given the variability in water distribution in soil throughout, particularly in large operations, having localized data on water stress can help farmers and researchers target specific crop areas to achieve more consistent and abundant crop yields.

Potential Alternative Uses for Hardware:

• Scaling design to measure stem/branch/fruit fluctuations and/or growth of various plants and trees, such as apple trees and cocoa beans
• Evaluating material expansion for things like metal in situations where the piece will be exposed to wide ranges of temperatures
• Research opportunities for viticulturists and horticulturists including possible extensions into plant communication and large-scale crop dynamics
• Tracking failure or displacement / relative location or motion

## 3 Design files

### 3.1 Design files summary

**Table:** 

**Design file name**	**File type**	**Open source license**	**Location of the file**
Long Body	STL/dwg	CERN	https://doi.org/10.5281/zenodo.4482948
Spring Hold	STL/dwg	CERN	https://doi.org/10.5281/zenodo.4482948
Vine Contact Mount	STL/dwg	CERN	https://doi.org/10.5281/zenodo.4482948
Rods	STL	CERN	https://doi.org/10.5281/zenodo.4482948
T Magnet Mount	STL/dwg	CERN	https://doi.org/10.5281/zenodo.4482948
Sandwich Grip (Upper and Lower)	STL/dwg	CERN	https://doi.org/10.5281/zenodo.4482948
Sensor Plate	STL/dwg	CERN	https://doi.org/10.5281/zenodo.4482948
Pelican Mount Top	STL	CERN	https://doi.org/10.5281/zenodo.4482948
Pelican Mount Bottom	STL	CERN	https://doi.org/10.5281/zenodo.4482948
LED Plug	STL	CERN	https://doi.org/10.5281/zenodo.4482948
Dendro.brd	BRD	CERN	https://doi.org/10.5281/zenodo.4482948
Dendro.sch	SCH	CERN	https://doi.org/10.5281/zenodo.4482948
SD_Output	INO	GNU GPL v3	https://doi.org/10.5281/zenodo.4482948
AS5311	H	GNU GPL v3	https://doi.org/10.5281/zenodo.4482948

**Long Body**: Central component of the frame of the dendrometer.

**Spring Hold**: Back piece of the frame that holds the stationary end of the spring.

**Vine Contact Mount**: Situated against the vine, holds up the dendrometer’s frame. Main attachment point between the vine and dendrometer.

**Rods**: Form a connection between T Magnet Mount and Sandwich Grip; there are two and each goes along the side of the vine without ever actually coming in contact with the plant.

**T Magnet Mount**: Mount for the magnet that provides a connection between the spring and vine motion.

**Sandwich Grip**: Part that holds the device in tension and is used against the far side of the vine. It is removable, and placement is adjustable. Used for installing the device and moving the spring/magnet in response to vine diameter fluctuations.

**Sensor Plate**: Holds the AS5311 magnetic sensor against the frame. Placed so that it is ∼ 0.3 mm from the suspended magnet on the SpringT.

**Pelican Mount**: This is a mount that is compatible with a 1120 pelican box - which is recommended for enclosing electronics. The mount works with a PVC pipe and has additional customization for use with trellis posts.

**LED Plug**: Provides an outlet from the pelican box to the outside environment to fasten the LED, which is used for LED indication system.

**Dendro.brd**: The EAGLE board file for the PCB

**Dendro.sch**: The EAGLE schematic file for the PCB

**SD_Output**: C++ file to operate the device and sensors

**AS5311**: Supporting C++ header file to operate the AS5311 sensor

*See Fig. 12 under “5.1.2 Mechanical Assembly” for a visual breakdown of the components

## 4 Bill of materials

Required materials outlined for the mechanical assembly; electronics hardware and enclosure; and 3D printed components.

### 4.1 Purchased materials for mechanical components

**Table:** 

**Designator**	**Component**	**Number**	**Cost per unit (USD)**	**Total cost (USD)**	**Source of materials**	**Material type**
Frame	Carbon Fiber Sheet (6″ x 6″ x 1/16″)	1/5of sheet	$4.99	$22.44	McMaster-Carr	Carbon Fiber
Frame	Carbon Fiber Sheet (6″ x 6″ x 1/4″)	$\frac{1}{10}$ of sheet	$4.94	$59.27	McMaster-Carr	Carbon Fiber
Extension Spring	Extension Spring (0.625″)	1^(E)^	$4.67	$14.01	McMaster-Carr	Stainless Steel
Rods	Carbon Fiber Rectangular Rod	$\frac{1}{10}$ of stock	$0.28	$2.65	ACP Composites	Carbon Fiber
4-40 Flathead Screws	Brass Slotted Flathead Screws 4-40 1/2″ Long	2^(D)^	$0.14	$6.82	McMaster-Carr	Brass
4-40 Phillips Screw	Brass Pan Head Phillips Screw 4-40 1/2″ Long	2^(D)^	$0.16	$8.22	McMaster-Carr	Brass
3-48 Slotted Screw	Brass Round Head Slotted Screw 3-48 1/2″ Long	4^(D)^	$0.24	$6.04	McMaster-Carr	Brass
2-56 3/8″ Long Roundhead Screw	Brass Round Head Slotted Screw 2-56 3/8″ Long	3^(D)^	$0.19	$6.46	McMaster-Carr	Brass
2-56 1/4″ Long Roundhead Screw	Brass Round Head Slotted Screw 2-56 1/4″ Long	1^(D)^	$0.05	$5.18	McMaster-Carr	Brass
2-56 Thumb Nut	Stainless Steel Thumb Nut 2-56	2	$3.54	$3.54	McMaster-Carr	Stainless Steel
2-56 Hex Nut	Brass Hex Nut 2-56	1^(D)^	$0.03	$3.13	McMaster-Carr	Brass
3-48 Hex Nut	Brass Hex Nut 3-48	4^(D)^	$0.13	$3.35	McMaster-Carr	Brass
Hose Clamp	¾”–1-¾” Stainless Steel Hose Clamp	1	$1.25	$1.25	Home Depot	Stainless Steel
Epoxy	Marine Epoxy	N/A	$0.10	$5.20	Home Depot	Non-specific
PVC Pipe	PVC Pipe 1-1/4″ OD 5ft Length	1	$8.04	$8.04	McMaster-Carr	Plastic
U Bolt	U Bolt	2	$4.85	$9.70	McMaster-Carr	Metal

### 4.2 Purchased materials for electronics and electronics set up

**Table:** 

**Designator**	**Component**	**Number**	**Cost per unit (USD)**	**Total cost (USD)**	**Source of materials**	**Material type**
Battery	Lipo Battery 3.7 V 2000mAh	1	$12.50	$12.50	Adafruit	Non-specific
Cable Glands PG7	Cable Glands PG7	2^(C)^	$0.80	$7.99	Amazon	Polymer
Weather- proofing	Conformal Coating	3 mL	$0.81	$12.95	Digikey	Acrylic
Pelican Case	Pelican 1120 Case	1	$31.95	$31.95	Amazon	Polymer
USB Micro-B to Micro-B	USB Micro-B to Micro-B (10″)	1	$14.35	$14.35	usbfirewire	Non-specific
Magnetic Sensor	AS5311 Linear Magnetic Sensor	1	$16.76	$16.76	Digikey	Non-specific
Magnet	Magnet (2 mm multipole 10 µm/m)	25 mm	$26	$317	Bomatec International Corp.	Non-specific
Temperature/ Humidity Sensor	SHT30 Weather Proof Temp/Humidity Sensor	1	$24.95	$24.95	Adafruit	Non-specific
Feather M0	Adafruit Feather M0 LoRa	1	$34.95	$34.95	Mouser	Non-specific
Hypnos Board	Hypnos Board	1	$16.00	$16.00	OPEnS Lab	Non-specific
μSD Card	SD Card (16 GB)	1	$6.19	$6.19	Amazon	Semiconductor
CAT5 cable	4 ft. CAT5 cable	1^(B)^	$1.55	$15.49	Amazon	Non-specific
Button	Button	1	$2.99	$2.99	Mouser	Non-specific
LED	Neopixel / LED	1^(A)^	$1.19	$5.95	Adafruit	Non-specific
PCB	PCB	1^(A)^	$2.20	$11.00	PCBWay	FR4
JST connector (2 pin)	JST connector (2 pin)	1^(B)^	$0.95	$9.50	Mouser	Non-specific
JST connector (3 pin)	JST connector (3 pin)	1^(B)^	$1.50	$15.00	Mouser	Non-specific
JST connector (4 pin)	JST connector 4 pin)	1^(B)^	$1.60	$7.99	Amazon	Non-specific
RJ45 connector	RJ45 connector	1^(B)^	$4.30	$43.03	Mouser	Non-specific
Coin cell	Coin cell battery	1^(B)^	$0.68	$6.80	Adafruit	Non-specific
Female Headers	Female Headers	2	$0.95	$1.90	Adafruit	Non-specific
Male Headers	Male Headers	2	$0.50	$1.00	Adafruit	Non-specific
Stacking Headers	Stacking Headers 12-pin and 16-pin	2	$1.25	$2.50	Adafruit	Non-specific
Heat Shrink	Exposed WiringProtection	1	$10.99	$10.99	Amazon	Polypropylene

*Any Feather M0 board will work; having the LoRa version is not necessary.

(A) Sold in packs of 5

(B) Sold in packs of 10

(C) Sold in packs of 20

(D) Sold in packs of 100

(E) Sold in packs of 3

### 4.3 3D printed components

**Table:** 

**Designator**	**Component**	**Grams and %Infill**	**Cost per unit (USD)**^A^	**Total cost (USD)**	**Source of materials**	**Material type**
Pelican Mount Top	Pelican Mount Top	120 g 20%	$2.91	$2.91	3DXTECH	Polymer
Pelican Mount Bottom	Pelican Mount Bottom	120 g 20%	$2.91	$2.91	3DXTECH	Polymer
LED Plug	⅝” LED Plug	1.26 g 25%	$0.07	$0.07	3DXTECH	Polymer

A. Approximate cost of 3D printed components (using Fusion3 F400 with 3DXTech ASA).

## 5 Build instructions

This section will cover the steps to construct and assemble the mechanical and electrical components of the dendrometer. We begin with machining the carbon fiber parts in a machine shop (Section 5.1.1) using the part drawings (Fig. 5, Fig. 6, Fig. 7, Fig. 8, Fig. 9, Fig. 10, Fig. 11) given, then assembling the mechanical components (5.1.2). To protect the electronics and connect them to the mechanical components of the device, modifications to a Pelican case will be made (Section 5.2). With the Pelican case prepared, the electronics hardware (Hypnos and Feather boards; custom PCB; and AS5311 and SHT30 sensors) will be set up (Section 5.3). The final assembly (Section 5.4) will connect the electronic and mechanical components of the dendrometer.

**Fig. 4:**
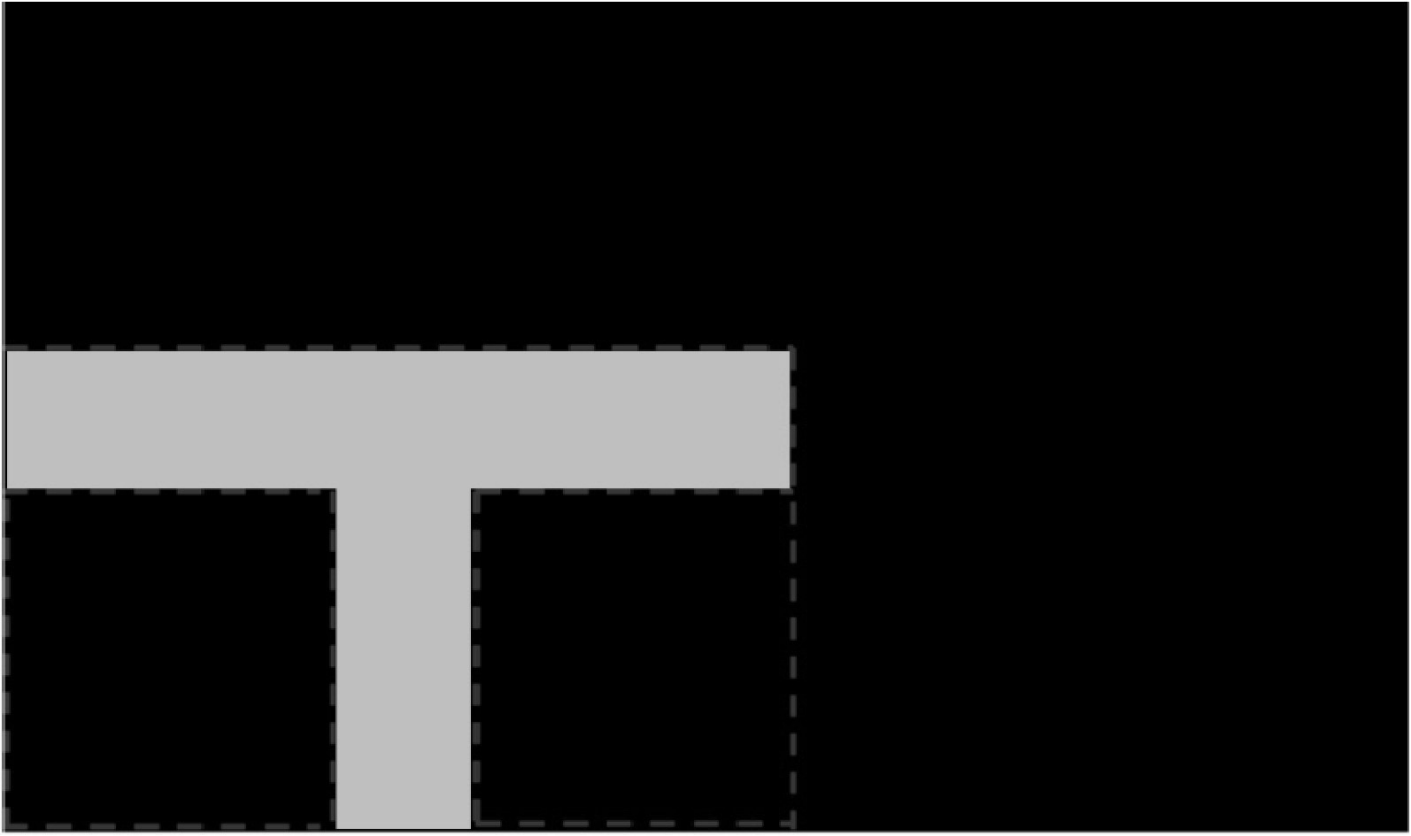
Cut lines (red dashed) for the T Magnet Mount (solid blue) on the 1/16″ thick carbon fiber sheet (not to scale). (For interpretation of the references to color in this figure legend, the reader is referred to the web version of this article.)

**Fig. 5:**
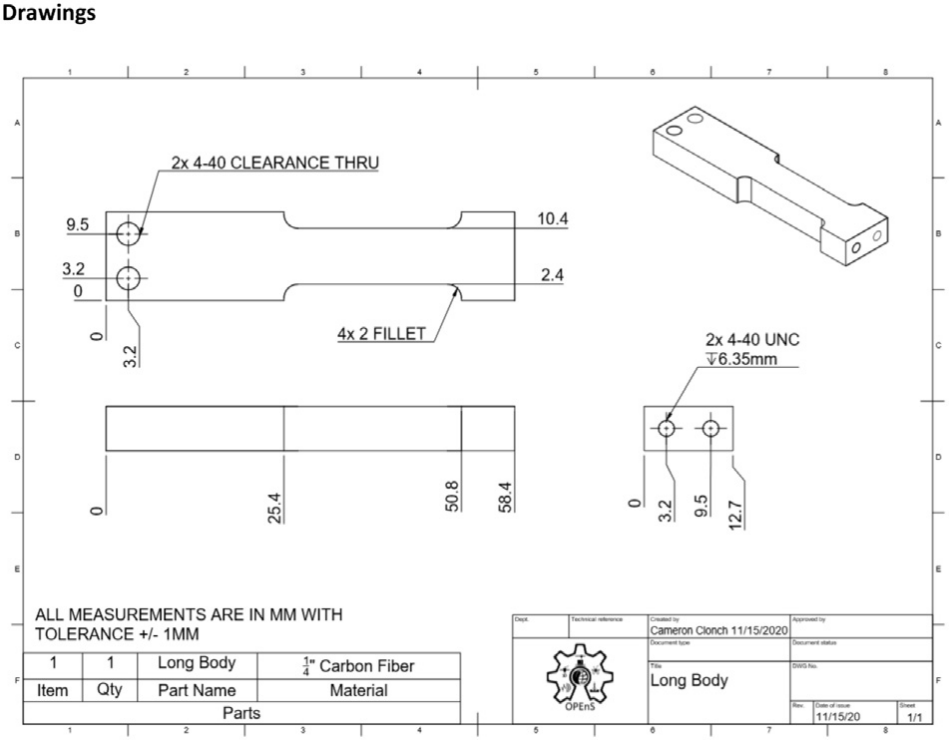
Long Body isometric CAD drawing. Counterclockwise from upper left: top, side, front, and isometric views.

**Fig. 6:**
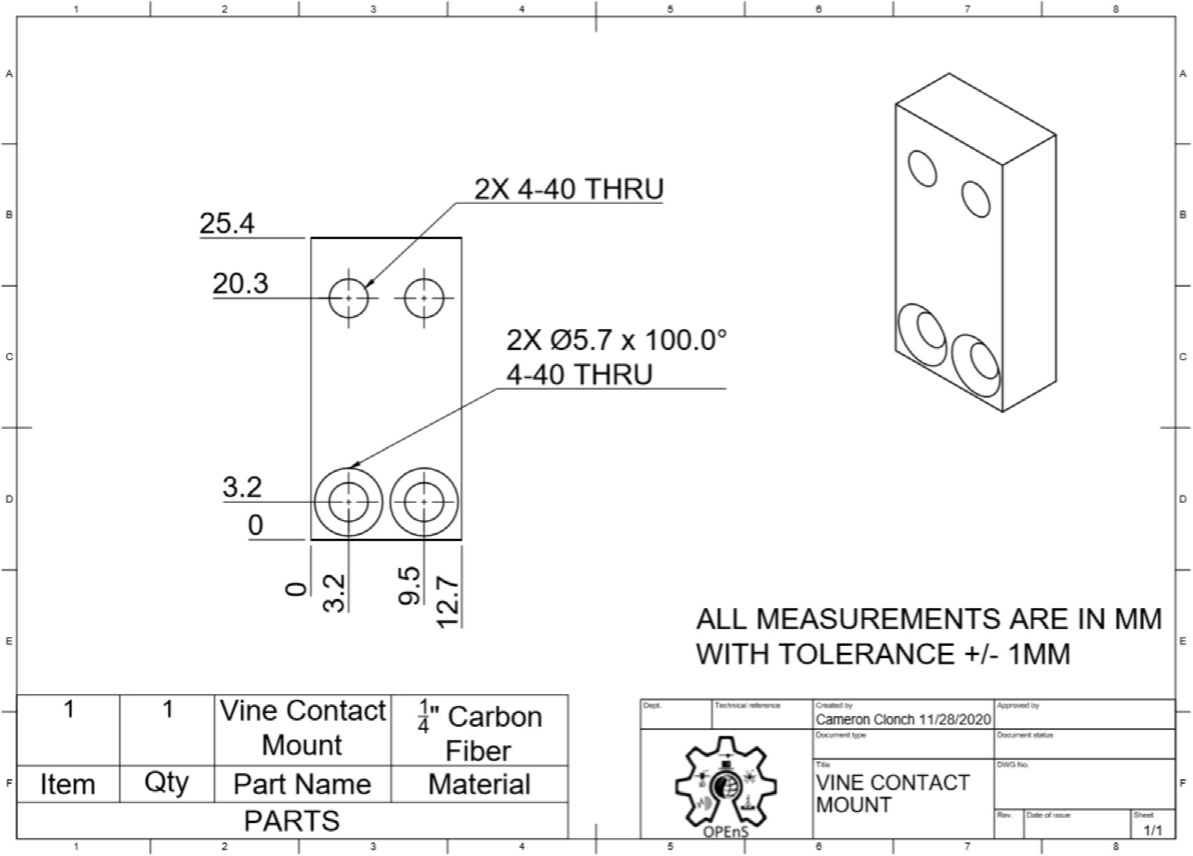
Vine Contact Mount CAD drawing (Left: front; and Right: isometric view).

**Fig. 7:**
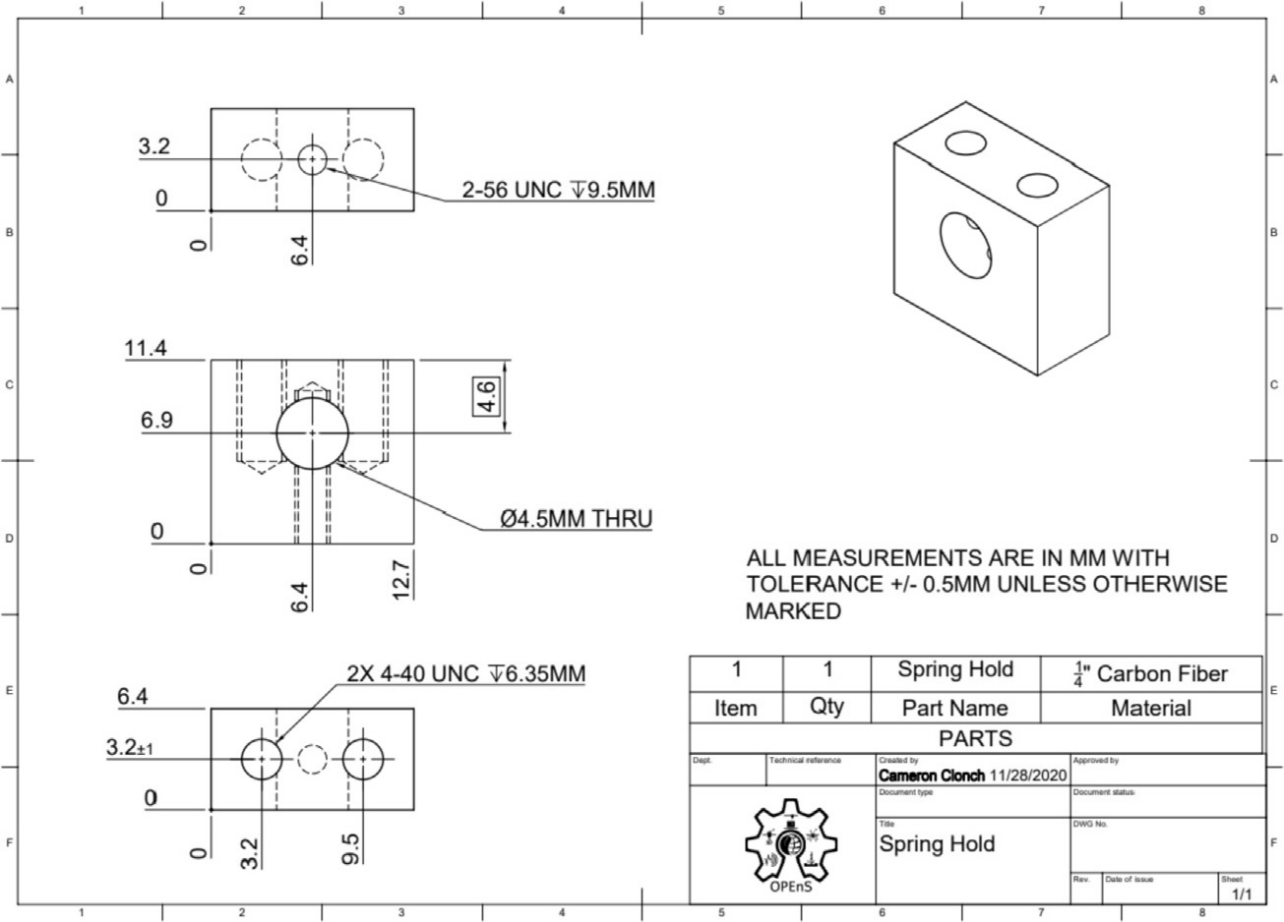
Spring Hold CAD drawing. Counterclockwise from upper left: top, front, bottom, and isometric view.

**Fig. 8:**
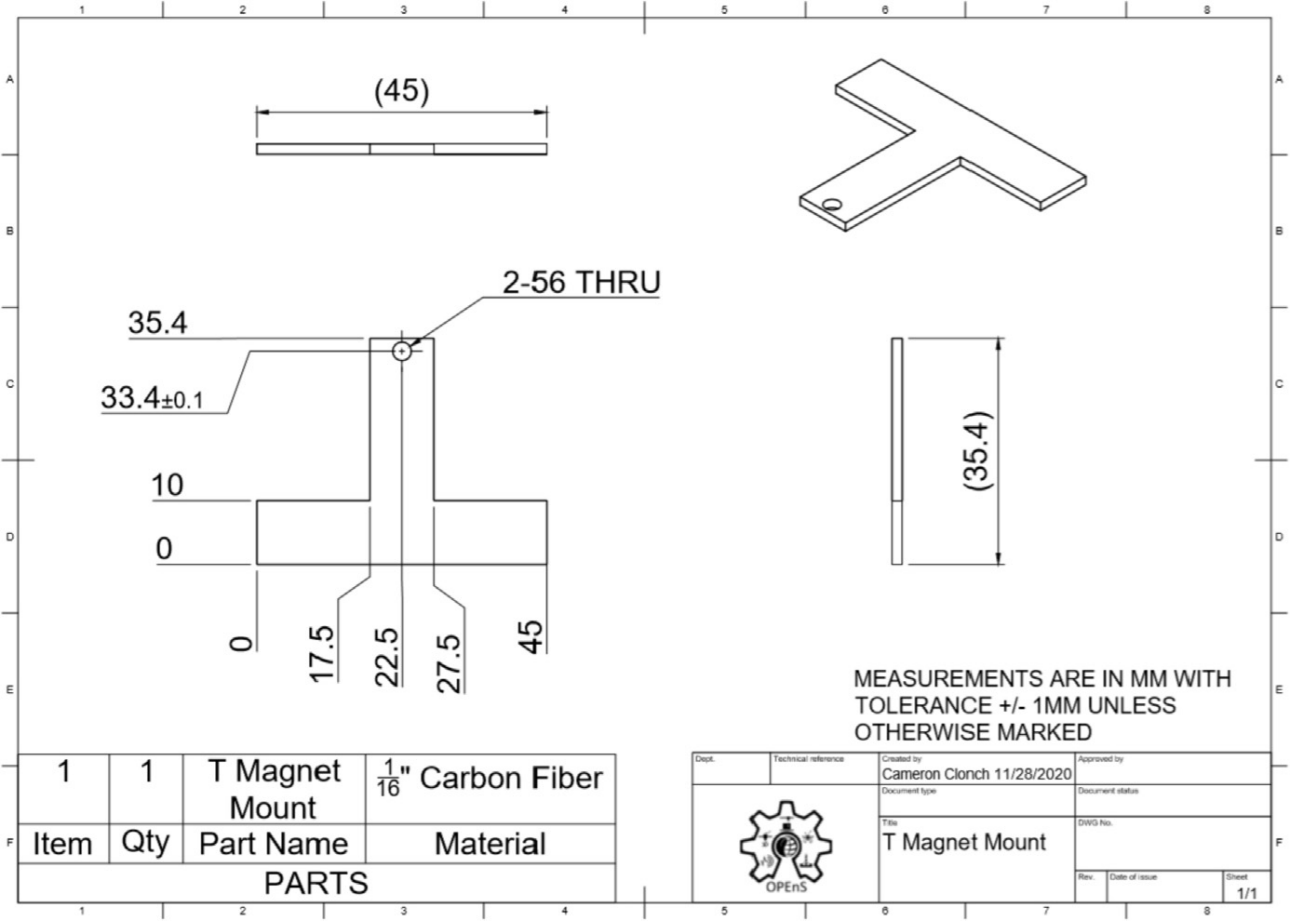
T Magnet Mount isometric CAD drawing. Counterclockwise from upper left: front, top, side, and isometric view.

**Fig. 9:**
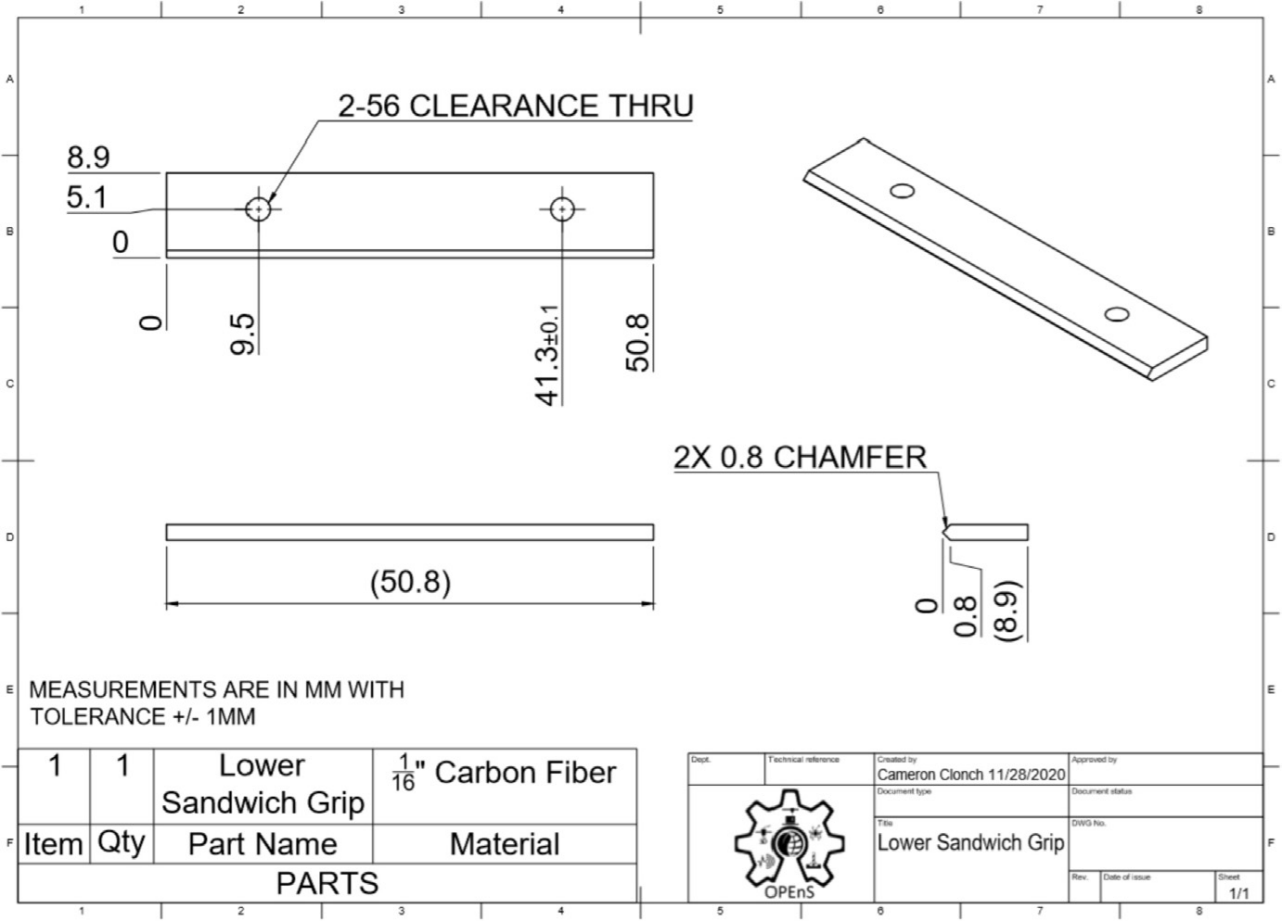
Lower Sandwich Grip CAD drawing. Counterclockwise from upper left: top, front, side, and isometric view.

**Fig. 10:**
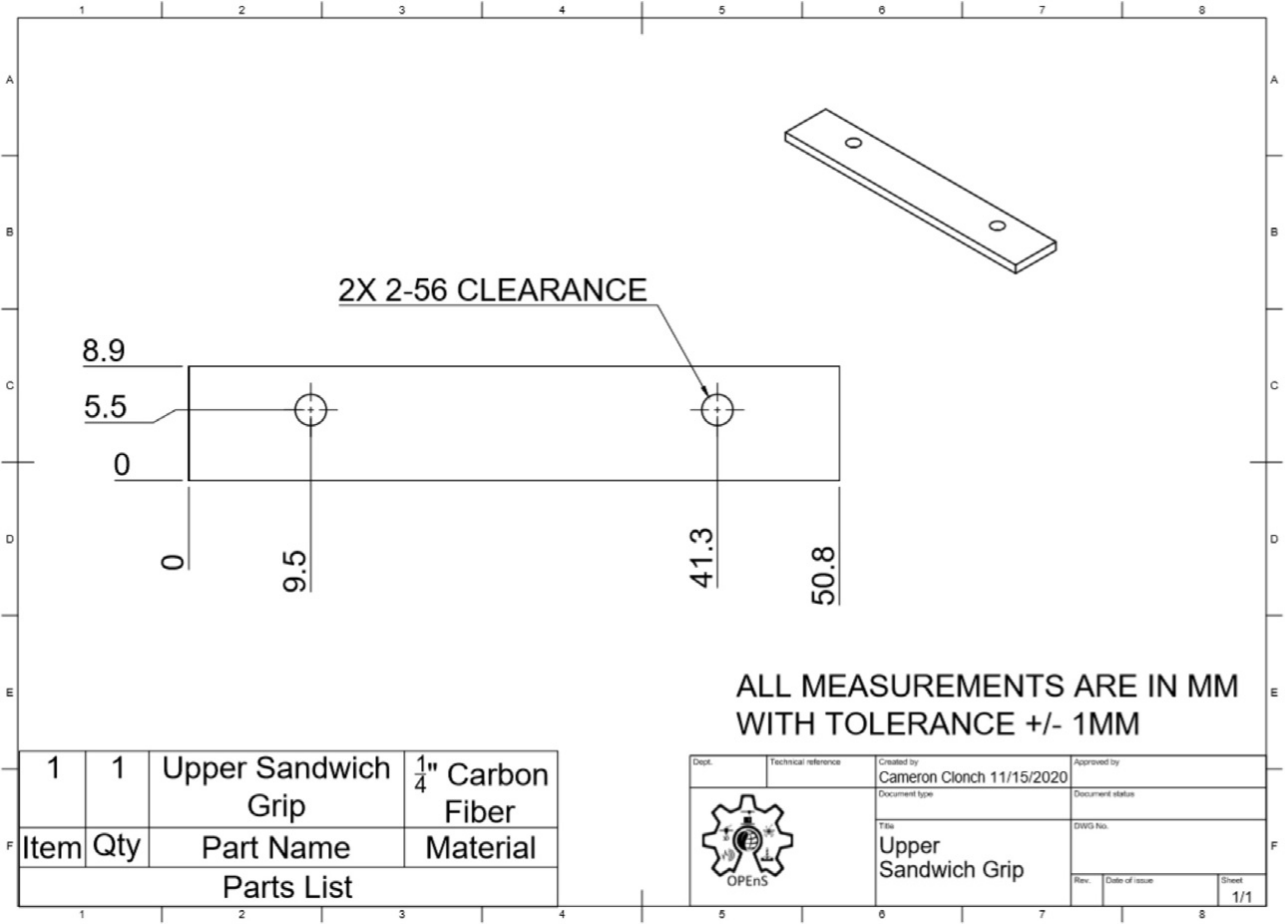
Upper Sandwich Grip CAD drawing (Left: top; Right: isometric view).

**Fig. 11:**
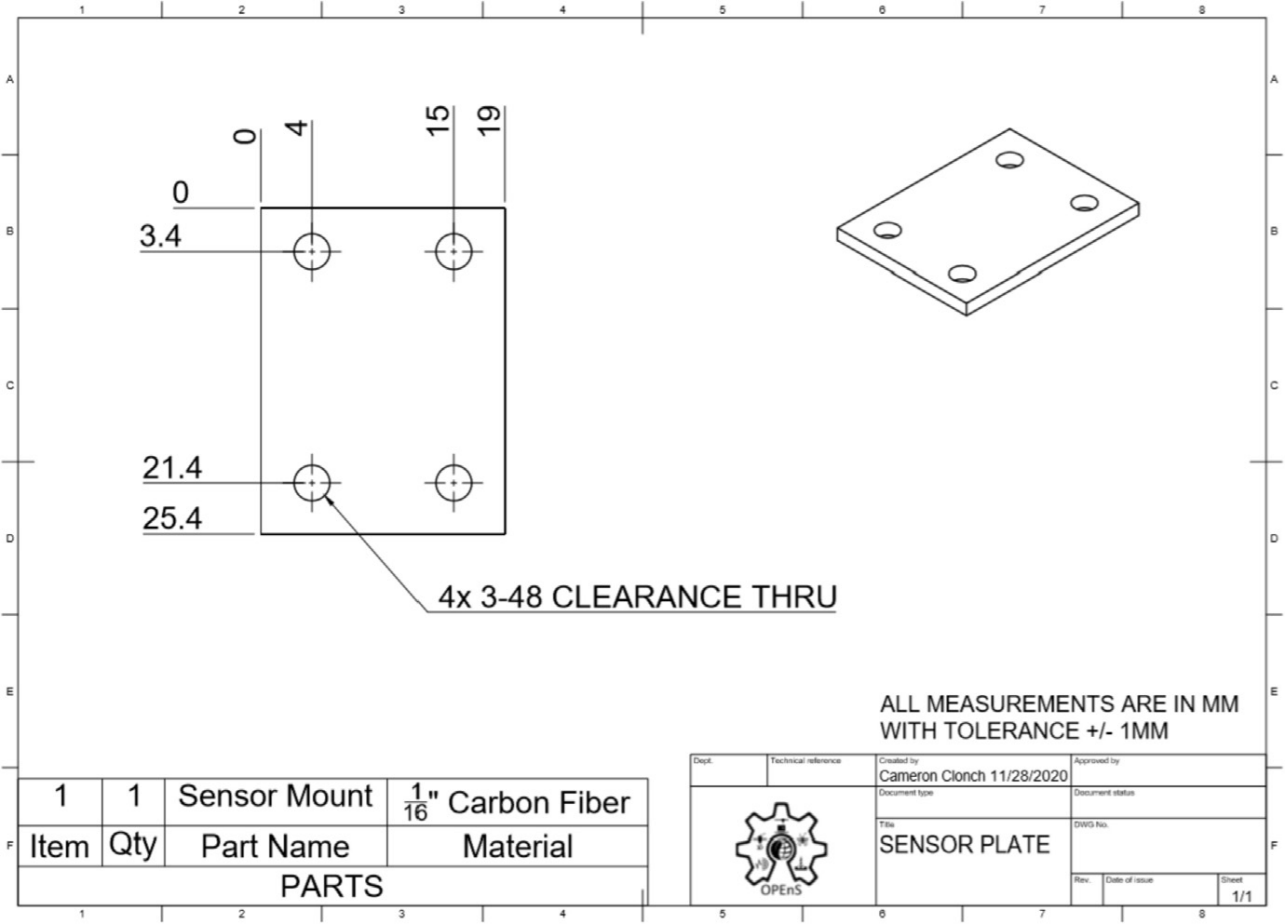
Sensor Plate CAD drawing. (Left: top; Right: isometric view).

**Fig. 12:**
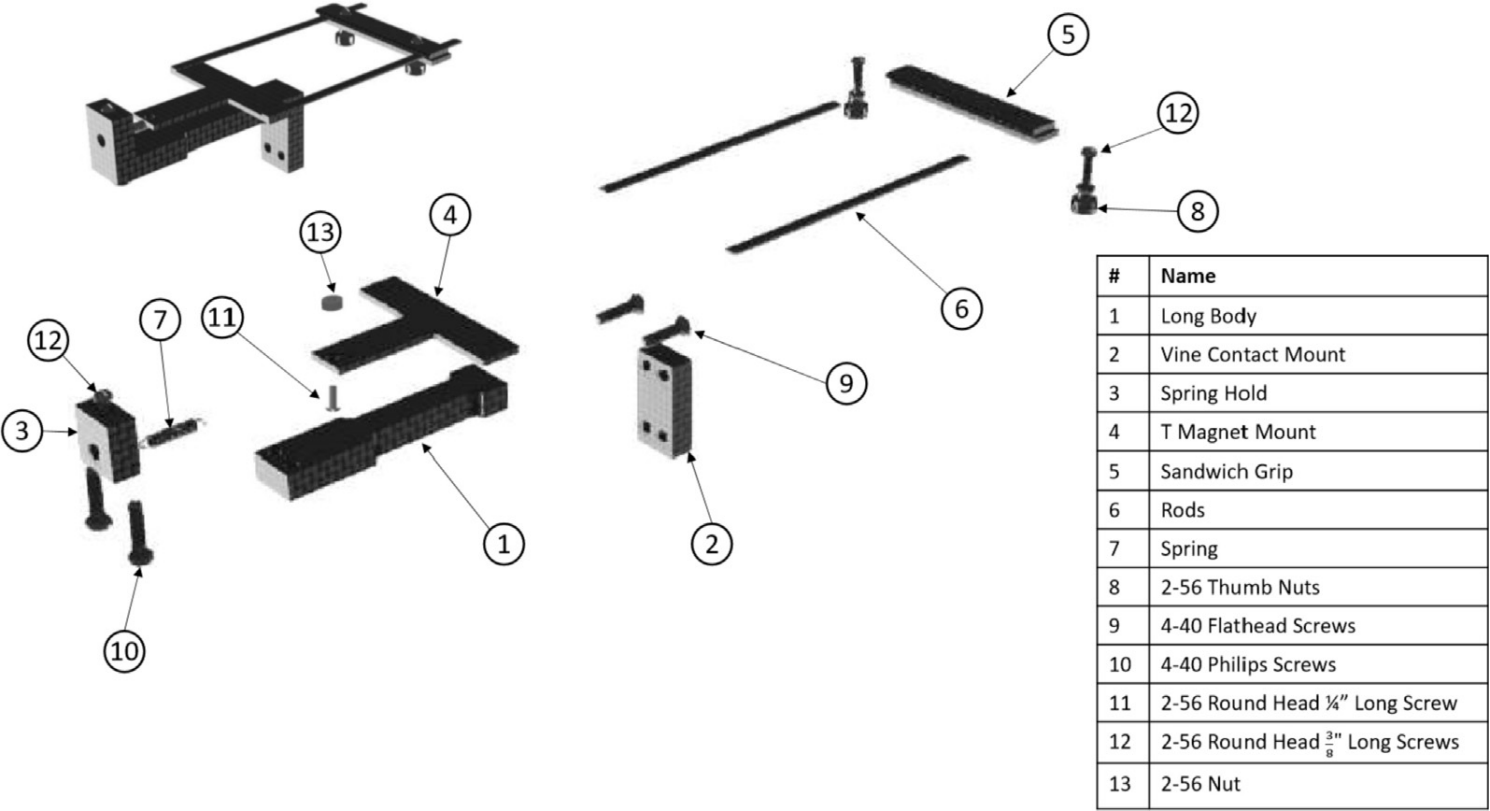
Exploded view of the dendrometer assembly with labeled components (excludes magnet and Sensor Plate as these come later in the assembly process).

### 5.1 Machined components

• Long Body (Fig. 5)
• Vine Contact Mount (Fig. 6)
• Spring Hold (Fig. 7)
• T Magnet Mount (Fig. 8)
• Sandwich Grips (Fig. 9,10)
• Sensor Plate (Fig. 11)
• Rods (do not require machining)

#### 5.1.1 Required tools

Milling machine (with drill chuck), bandsaw, threading tool, digital calipers, file

#### 5.1.2 Machining considerations

Carbon fiber tends to dull tools and must be cut slowly to avoid material fracture. However, this material has been specifically selected (and must not be exchanged for another) because it has an extremely low coefficient of thermal expansion so the dendrometer will remain accurate regardless of ambient diurnal temperature fluctuations.

There are also safety procedures that should be followed while using certain machinery; see band saw safety considerations [20] and milling safety considerations [21] for more information.

Recommended steps for machining

* Future iterations will use a waterjet cutter to machine the dendrometer into just three components and significantly reduce drilling.

General Tips for success:

• Use digital readout to help meet tolerances
• Ensure each piece is well supported to avoid fracture
• Drill down slowly - pecking may be helpful
1. Start by using the vertical bandsaw to cut out pieces of the ¼” thick carbon fiber stock for each element designated below.
  a. Slowly cut a strip that is 13 mm × 101 mm
  b. Cut to length the Long Body (Fig. 5, 58.4 mm), Vine Contact Mount (Fig. 6, 25.4 mm), and Spring Hold (Fig. 7, 11.4 mm)
  c. Measure with calipers, make additional cuts if necessary
  d. Set aside
2. With the vertical bandsaw, make cutouts from the 1/16″ carbon fiber stock.
  a. Cut a 8 mm × 51 mm strip for the two pieces of the Sandwich Grip (Fig. 9,10), measure, cut in half lengthwise: create two, 8 mm × ∼25.5 mm pieces
  b. Next, follow drawing dimensions to cut T Magnet Mount (Fig. 8)
    i. Cut rectangle matching largest width and length dimensions (35.4 X 45 mm), then cut out two smaller rectangles (17.5 × 25.4 mm) from this cutout (starting from bottom two corners) to make the T shape (Fig. 4)
  c. Cut out Sensor Plate (Fig. 11) using drawing dimensions (25.4 × 19 mm)
3. Switch to milling machine to make holes in the cutouts based on part drawings
  a. Install drill chuck in the quill
  b. Long Body (Fig. 5)
    ii. Use #32 drill bit, make the 4-40 clearance holes
    iii. Use #43 drill bit for holes marked 4-40 UNC – these will be tapped later
  c. Vine Contact Mount (Fig. 6)
    iv. Use a #32 drill bit to make all four holes
  d. Install countersink tool into drill chuck to countersink holes designated on drawing
  e. Spring Hold (Fig. 7)
    v. Use #43 drill bit for holes marked 4-40 UNC – these will be tapped later
    vi. Use #50 drill bit for 2–56 tapped hole – will be tapped later
    vii. Use #25 drill bit for the 4.5 mm through hole
  f. Make sure to pay attention to the tolerance of the Spring Hold (as noted on the drawing, dimensions must be within 0.5 mm of marked values)
  g. T Magnet Mount (Fig. 8)
    viii. Use #43 drill bit for the hole
  h. Sandwich Grip (Fig. 9,10)
    ix. Use #43 drill bit to create all holes on both the upper and lower Sandwich Grip
  i. Sensor Plate (Fig. 11)
    x. Use #42 drill bit for all holes
4. Using an end mill, cut the slots in the Long Body (Fig. 5)
a. Each cut should have a depth of 2.4 mm and be approximately 25.4 mm long
b. Must be well supported
c. Screws will sit in the milled out portions of the Long Body, allowing small adjustments to be made for best magnet/sensor alignment
5. Tap holes based on part drawings (shown below)
  a. Long Body (Fig. 5)
i. Two holes on the end (see drawing) require 4–40 UNC tap
  b. Spring Hold (Fig. 7)
ii. The two holes marked 4–40 UNC must be tapped with a 4–40 UNC tap
iii. The hole marked 2–56 UNC must be tapped with a 2–56 tap
6. Cut two, 90 mm long, pieces from the Carbon Fiber Rectangular Rods stock using strong clippers or bandsaw to create the Rods
7. On the Lower Sandwich Grip (Fig. 9), use a file to create chamfer on designated edge (refer to drawing)
a. Creates pointed end to help secure against vine upon installation
b. Finish with sand paper to make the surface of the edge rough (creates more friction between Sandwich Grip and vine when on a plant)

### 5.2 Drawings

#### 5.2.1 Mechanical assembly

1. Using two 4-40, ½” long flathead screws (Fig. 12: 9), fasten the Vine Contact Mount (Fig. 12: 2) to the Long Body (Fig. 12: 1) using the countersunk holes with the countersunk part on the outside.
  a. Note: 3-48 screws can be used in place here, but holes must be drilled and tapped on respective pieces accordingly.
  b. Screws should be flush with, or below, the front face of the Vine Contact Mount (Fig. 13).

**Fig. 13:**
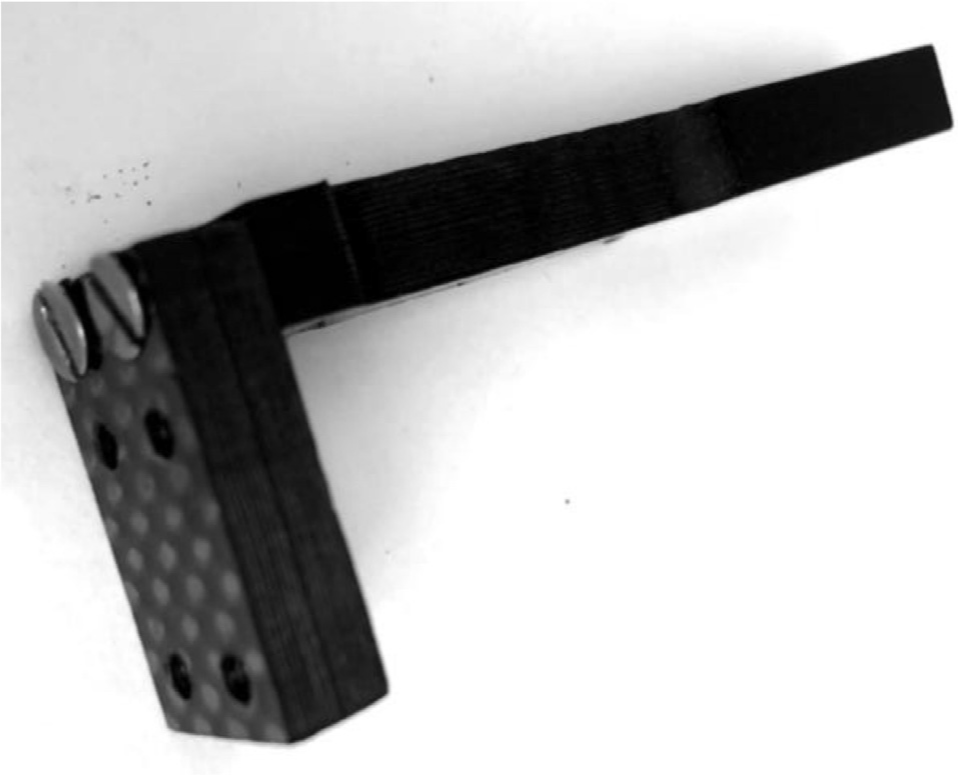
Vine Contact Mount connected to Long Body with two flathead screws (this Vine Contact Mount has two unnecessary additional holes in the middle).
2. Using two 4-40 ½” long pan-head Phillips screws (Fig. 12: 10), secure the bottom of the Spring Hold (Fig. 12: 3) to the Long Body (Fig. 12: 1) so that the piece forms an S-like shape (Fig. 14).
  a. Note: 3-48 screws can be used here as well (if modifications in drilling operations were made).

**Fig. 14:**
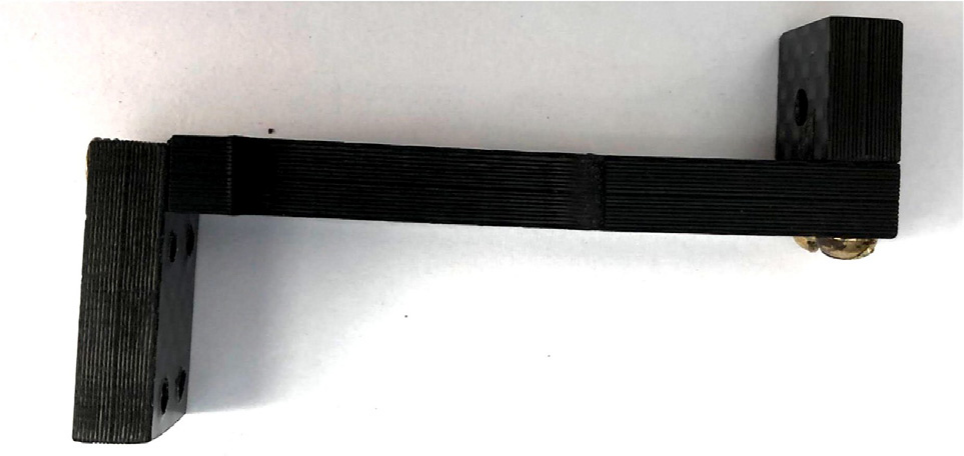
Spring Hold (right) attached to the Long Body (center). The Vine Mount (left) is attached from the previous step.
3. Place a ⅜” long 2-56 screw (Fig. 12: 12) into the 2-56 threaded hole on the exposed end of the Spring Hold (Fig. 12: 3); before it is fully screwed in, slip one circular hook end of the spring (Fig. 12: 7) into the 4.5 mm hole on the front face of the Spring Hold and continue to screw, allowing the screw to go through the spring’s hook, securing the spring on this fixed end of the dendrometer (Fig. 15).

**Fig. 15:**
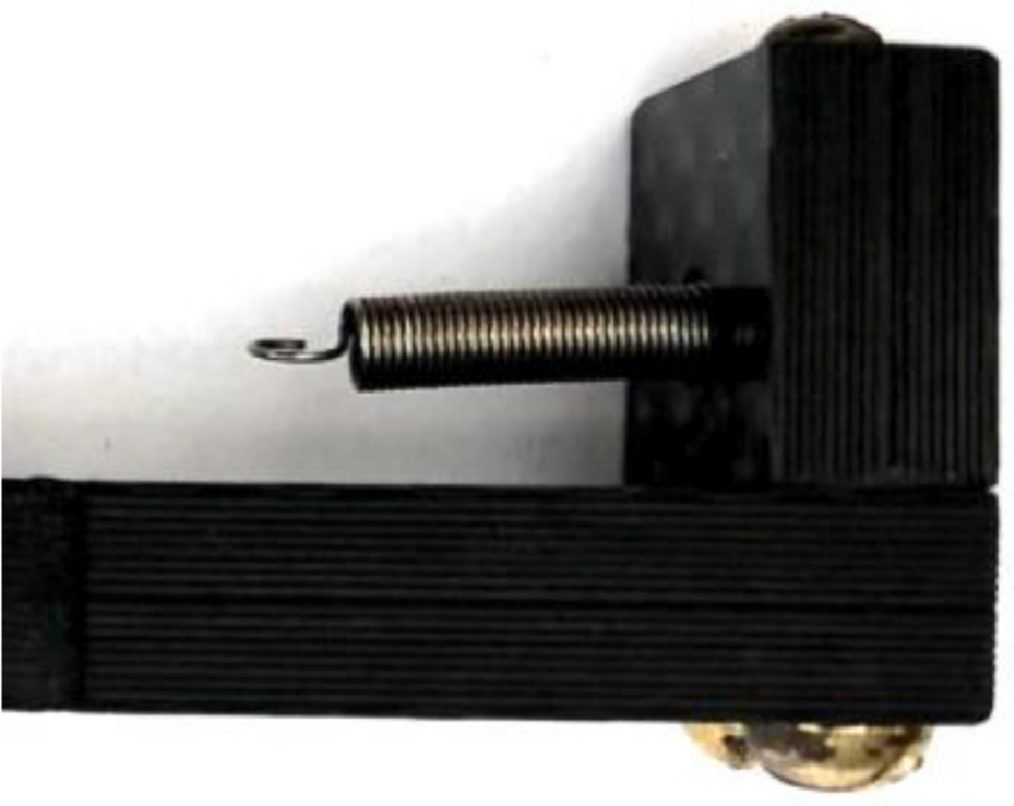
Spring extends out from the 4.5 mm hole, where it is hooked around a screw inside the Spring Hold. Spring is above the Long Body.
4. On both ends of the T Magnetic Mount (Fig. 12: 4), epoxy a Rod on top so that they are perpendicular to the arm and the ends are flush with the edges of the T Magnet Mount.
a. May want to use grid paper or a right angle to ensure that the rods are parallel to each other and perpendicular to the T Magnet Mount arm.
b. Clamp down rods (binder clips work well) and allow epoxy to set for several hours or overnight (Fig. 16).

**Fig. 16:**
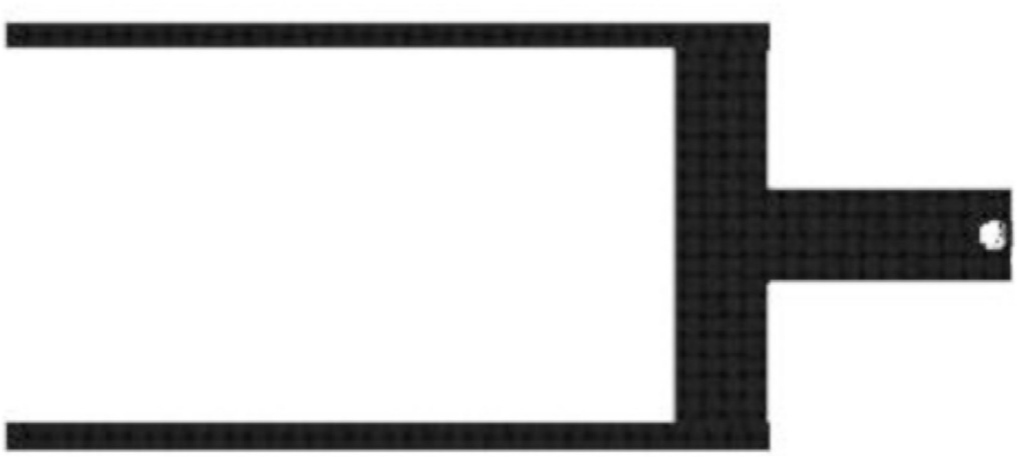
Image showing proper orientation of the T Magnet Mount and Rods relative to one another.
5. Insert a #2 screw (Fig. 12: 11) through the circular hook on the open end of the spring, then put the screw (with the spring on) through the hole on the T Magnet Mount (Fig. 12: 4); use a 2-56 nut (Fig. 12: 13) to fasten the spring to the T.
  a. The nut should be on the side of the T furthest from the Long Body.
  b. Ensure that the spring is collinear with the stem of the T when fastened.
  c. Check that the spring clears the end of the T stem and the hook is well sandwiched between the T stem and the screwhead (Fig. 17).

**Fig. 17:**
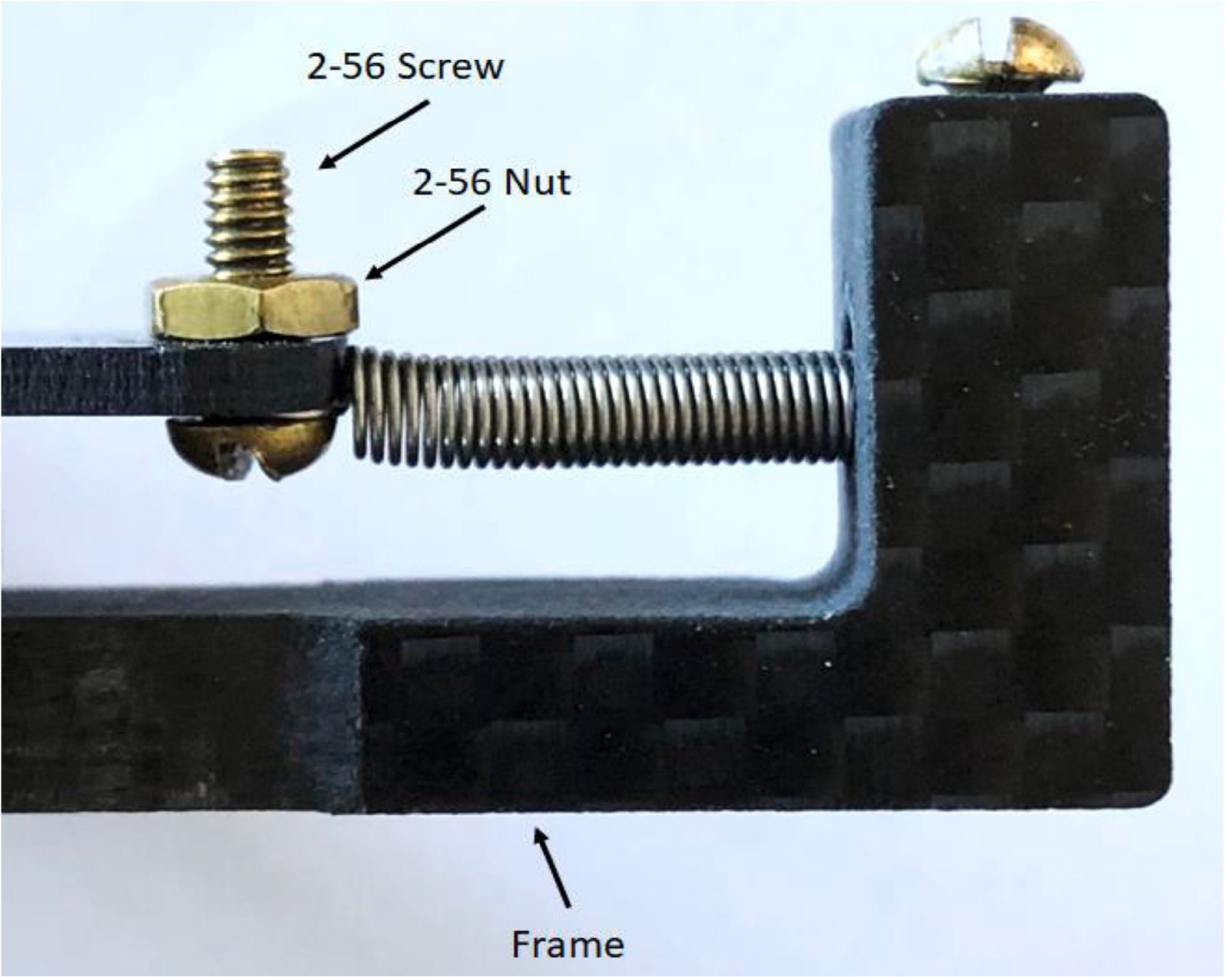
The spring has a hook on either end; one side is connected to the Spring Hold (right) from a previous step. The left end of the spring is hooked around the screw shown, being sandwiched between the underside of the screw head and the T Magnet Mount (it is on the nearside of the T Magnet Mount and held in place by this pressure).
i. This may require stretching the spring slightly.
ii. If spring still overlaps the T Magnet Mount, file the end (carefully so the hole remains intact) until it no longer creates a bump; the spring can be against the end of the T, but not on the same plane as the nut or screw head.
6. Using clippers, cut a piece from the magnet strip that is approximately 20 mm long. Note that if the dendrometer being built has been scaled to accommodate larger stems or vines, the magnet will need to be longer as well so the device can measure larger-valued oscillations and retain long-term deployment suitability.
7. With the epoxy (or adhesive if already included on the magnet), apply a thin, even layer to the stem of the T Magnet Mount (Fig. 12: 4) and carefully place the magnet on top, aiming to get it as centered as possible.
  a. Length of the magnet should be collinear with the stem of the T.
  b. Place the magnet so that it just clears the screw holding the spring down (Fig. 18).

**Fig. 18:**
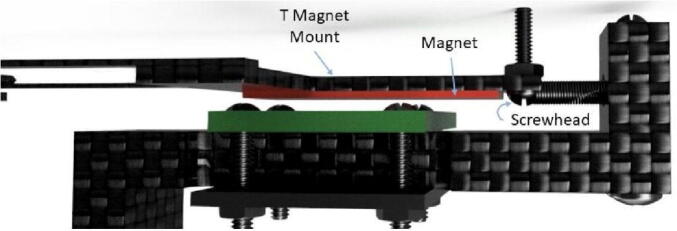
The magnet (red) is placed collinear with the T Magnet Mount stem, with one end next to the 2–56 screw that clamps the hook of the spring against the T Magnet mount. (For interpretation of the references to color in this figure legend, the reader is referred to the web version of this article.)
8. Connect the upper and lower components of the Sandwich Grip (Fig. 12: 5) using two, ⅜” long 2–56 screws (Fig. 12: 12) and thumb nuts (Fig. 12: 8).
a. The orientation of the screws and the thumb nuts on the sandwich grip is unimportant.
b. Tighten the two pieces together.

### 5.3 Pelican case modifications

To allow for individual, waterproof pass through of sensor cables (AS5311, SHT30), USB access, and LED indication system, five holes need to be drilled and filled.

**Fig. 19:**
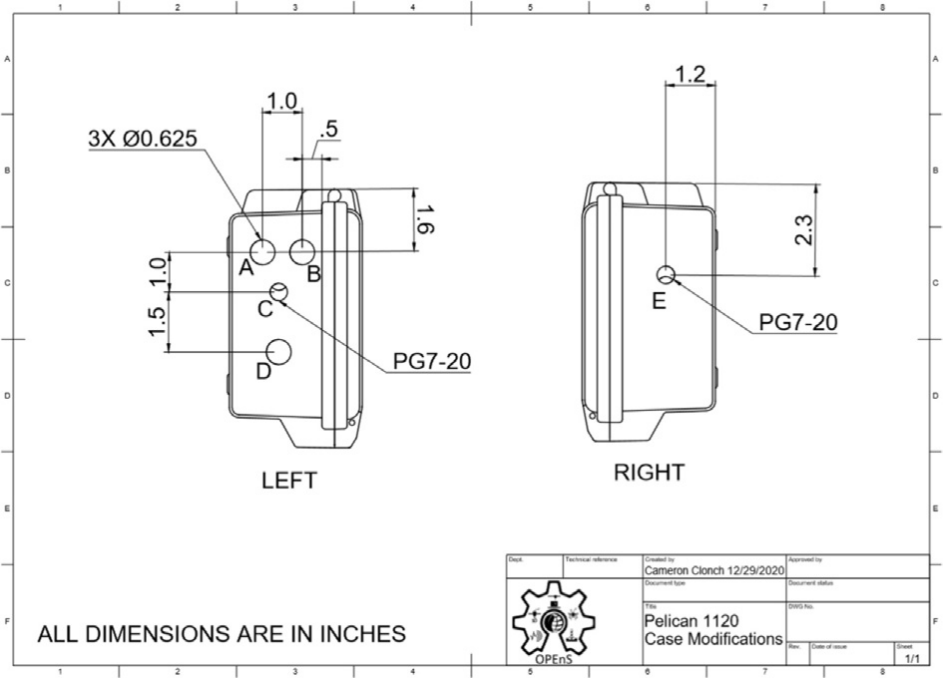
CAD drawing of modifications to Pelican Case.
3) Run the cable for the SHT30 through the cable grip in hole E (Fig. 19: right side). The metal sensor end should be outside the case and the wiring/electronics will be stored inside (Fig. 20).

1) Remove and discard the foam from the pelican case.
2) For the sensor cables, drill two 7/16″ holes (Fig. 19: C, E), one on each of the two opposing sides shown in Fig. 19, and tap the holes with PG7-20 threads into the Pelican Case according to the figure. Thread PG7 cable grips into the Pelican case.

**Fig. 20:**
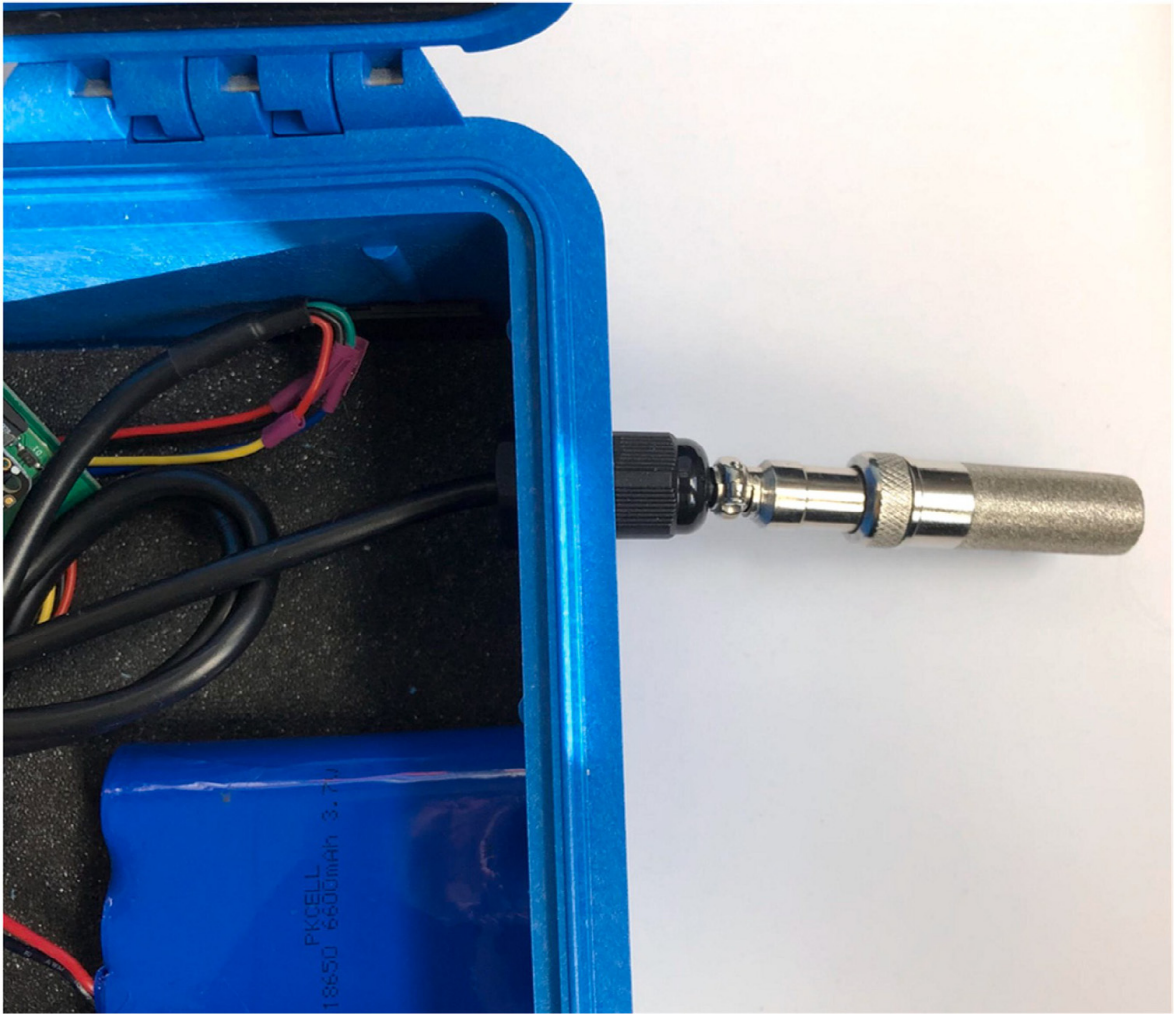
The SHT30 with the sensor end on the outside (protruding from the black cable grip) and the cable on the inside of the Pelican Case.
4) For the USB port, drill hole D (⅝-inch diameter) based on Fig. 19. Thread in the USB extension and place cap over the USB port (Fig. 21).

**Fig. 21:**
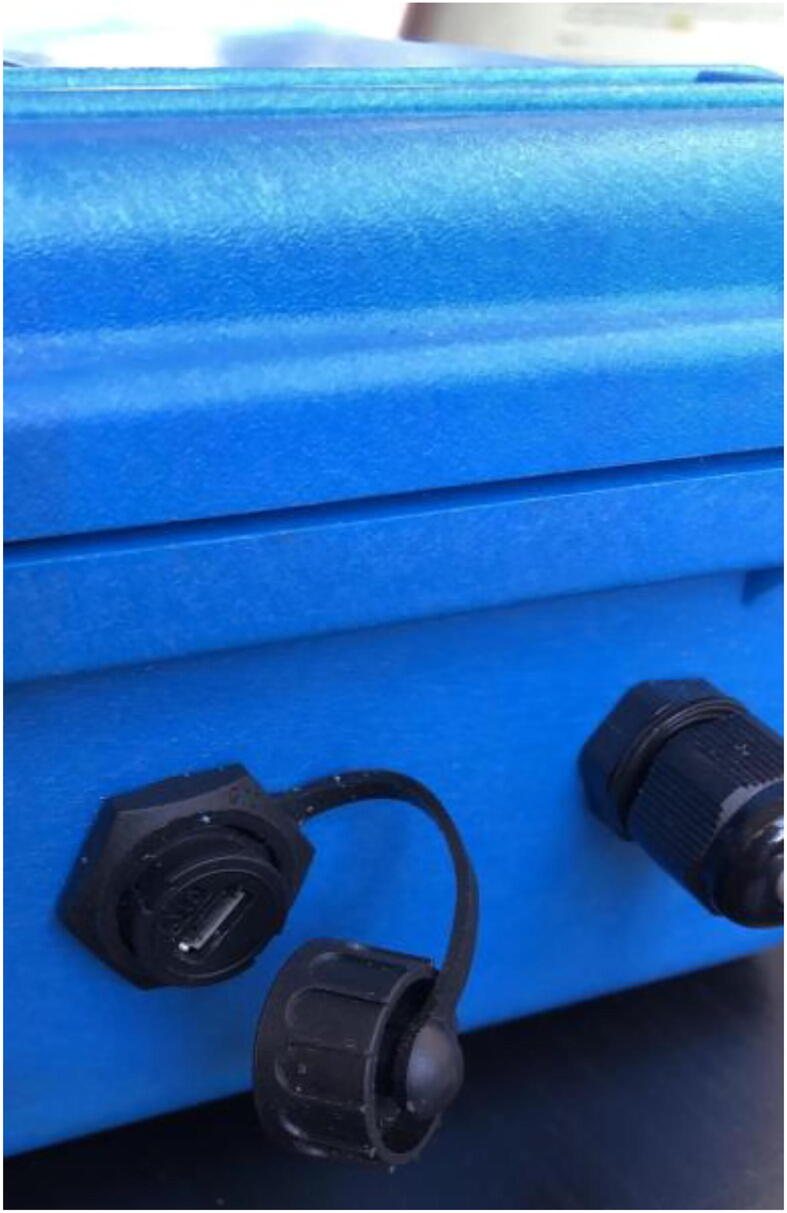
The USB port (with the cap off) on the left side of the Pelican Case.
5) Drill two more ⅝-inch diameter holes (Fig. 19: A, B) above the rest of the holes on the left side of the case: one for the LED Plug and the other for the button. Thread the button in one hole (use the given nut to secure it in place on the inside of the case) and the ⅝-inch 3D-printed plug in the other (Fig. 22).

**Fig. 22:**
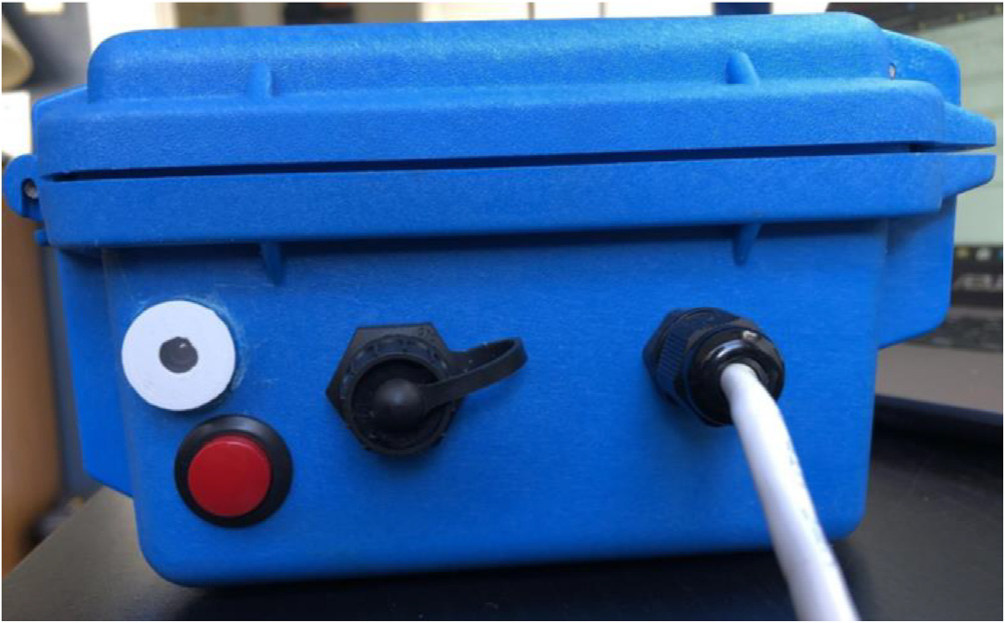
The completed left side of the Pelican Case with LED plug, button, micro-USB port, and PG7 cable gland (the CAT5 cable in the cable grip of hole D will be implemented later).

Note: These are only suggestions for how to set up the Pelican Box; other hole configurations work as well if desired.

### 5.4 Electronics hardware setup

Required Tools

‐ Soldering iron
‐ Solder

Review the Bill of Materials required for this prototype, then gather all of the materials necessary for the construction

Feather / Hypnos:

1. Set up at the soldering station with a Adafruit Feather M0 LoRa, a Hypnos board, two sets of male headers and one set of female headers as specified in the BOM
2. On the Hypnos Board, solder one set of female headers to the Feather Rail and a set of male headers on the sensor rail pointed downward (Fig. 23)
  a. Note that stacking headers can be used in place of male headers on the sensor rail (as shown in Fig. 23)

**Fig. 23:**
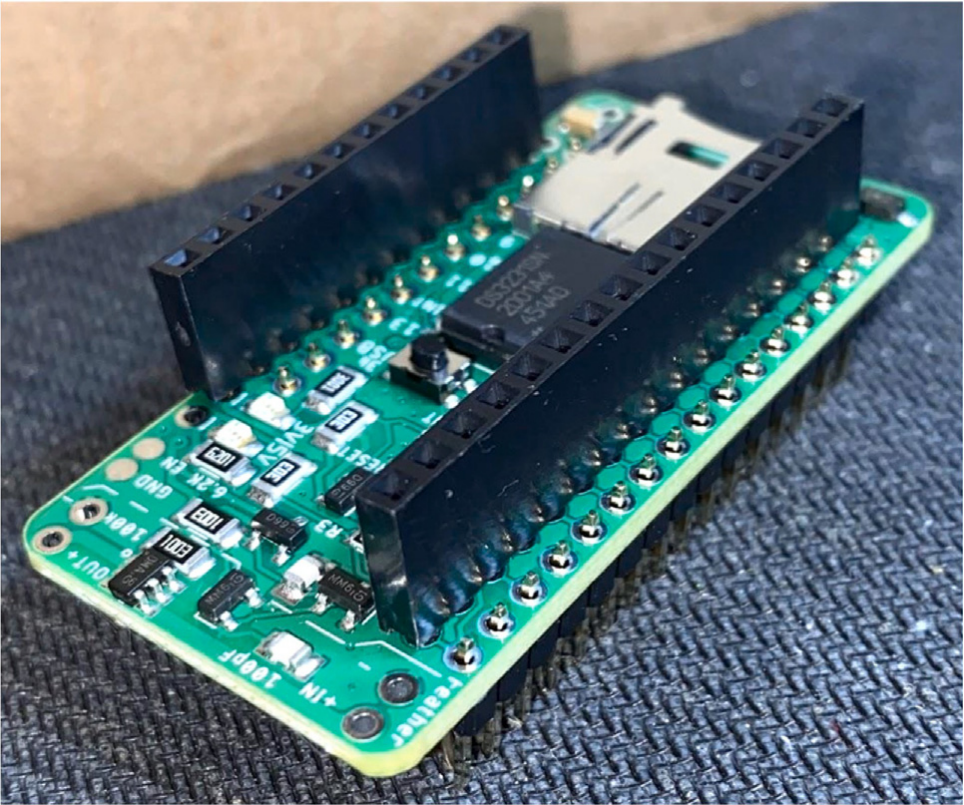
Hypnos with a set of stacking headers and male headers.
3. Solder male headers to the Adafruit Feather M0 LoRa pointed downward (Fig. 24)

**Fig. 24:**
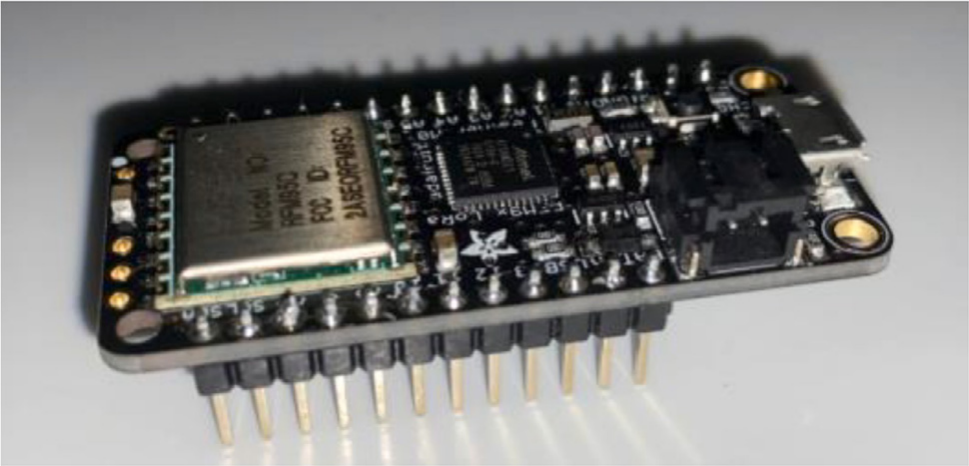
Feather board with male headers.

**Fig. 25:**
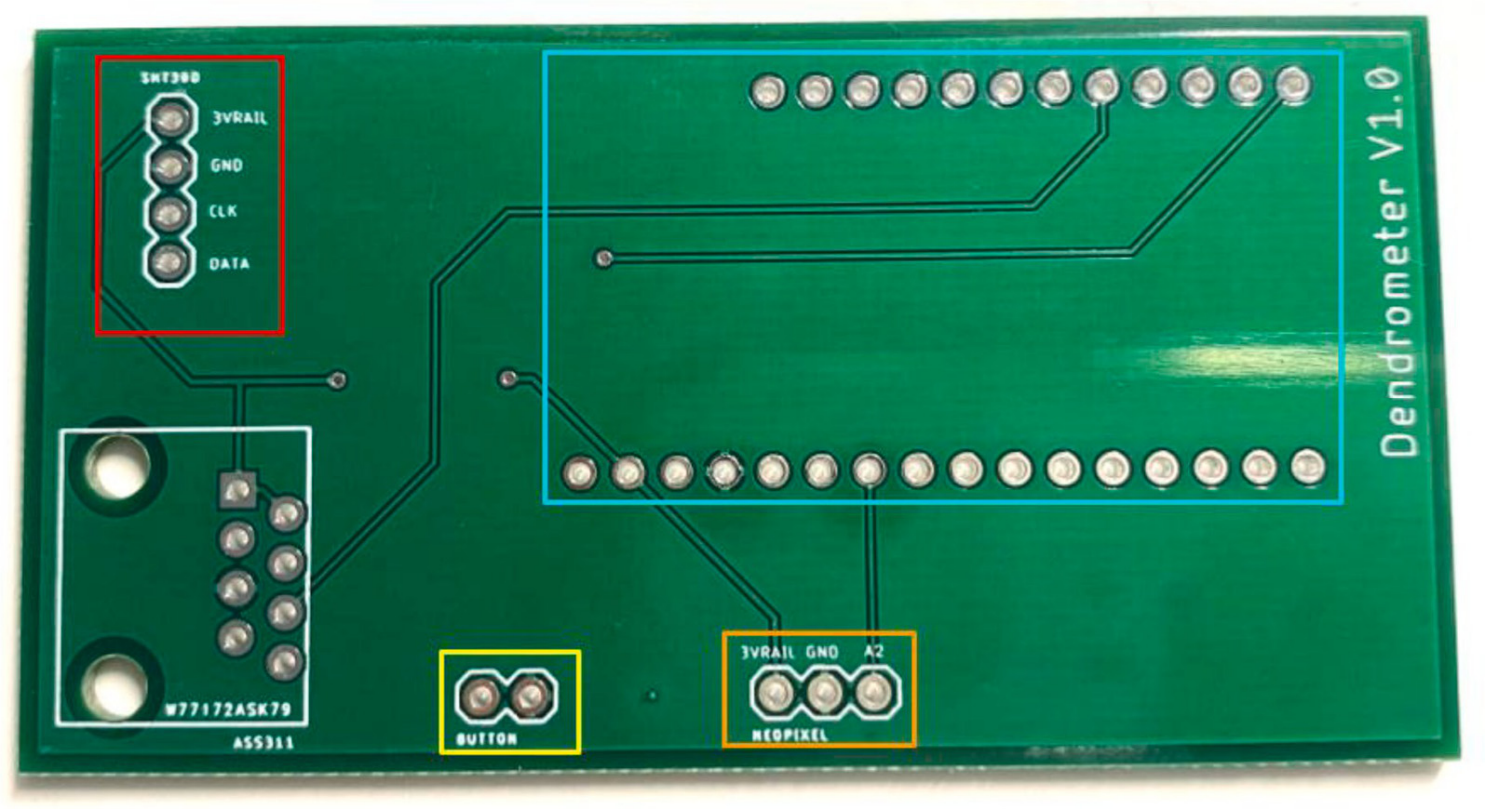
PCB Before JST Connectors and RJ45 Connector. The red box indicates the 4 pin JST for the SHT30, blue box shows the Feather M0 footprint, yellow is the 2 pin JST for the button, orange is the 3 pin JST for the Neopixel LED, and the white box shows the RJ45 connector. (For interpretation of the references to color in this figure legend, the reader is referred to the web version of this article.)

PCB:

1. On the custom PCB, solder female header pins in the Feather footprint, JST connectors (2 pin, 3 pin, and 4 pin), and the RJ45 connector (Fig. 25, Fig. 26).

**Fig. 26:**
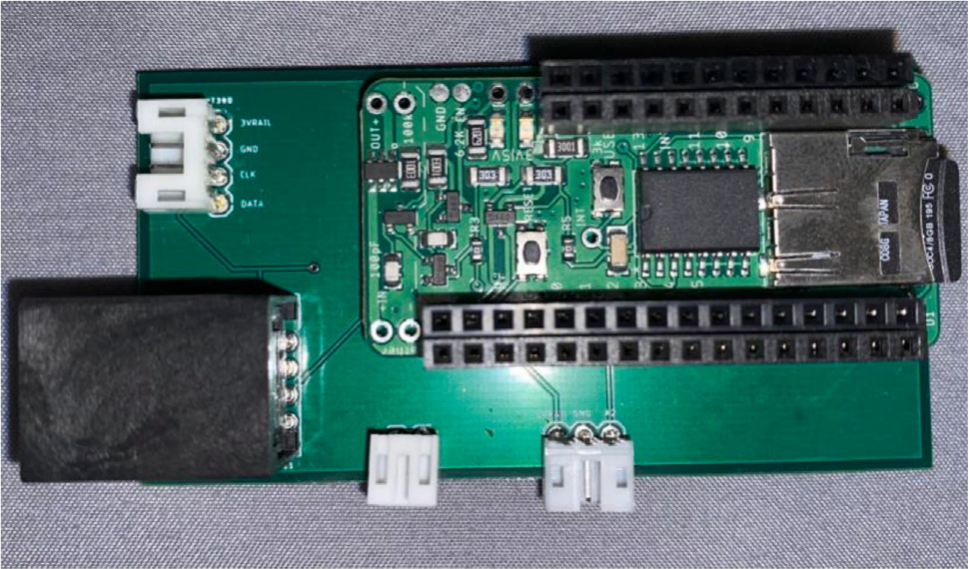
Completed PCB with JST Connectors, RJ45 Connector, and Hypnos.
2. Insert a microSD card and coin cell battery into the Hypnos board so the RTC can continue to run during the sampling period.
3. Stack the Feather M0 on top of the Hypnos board (Fig. 27).

**Fig. 27:**
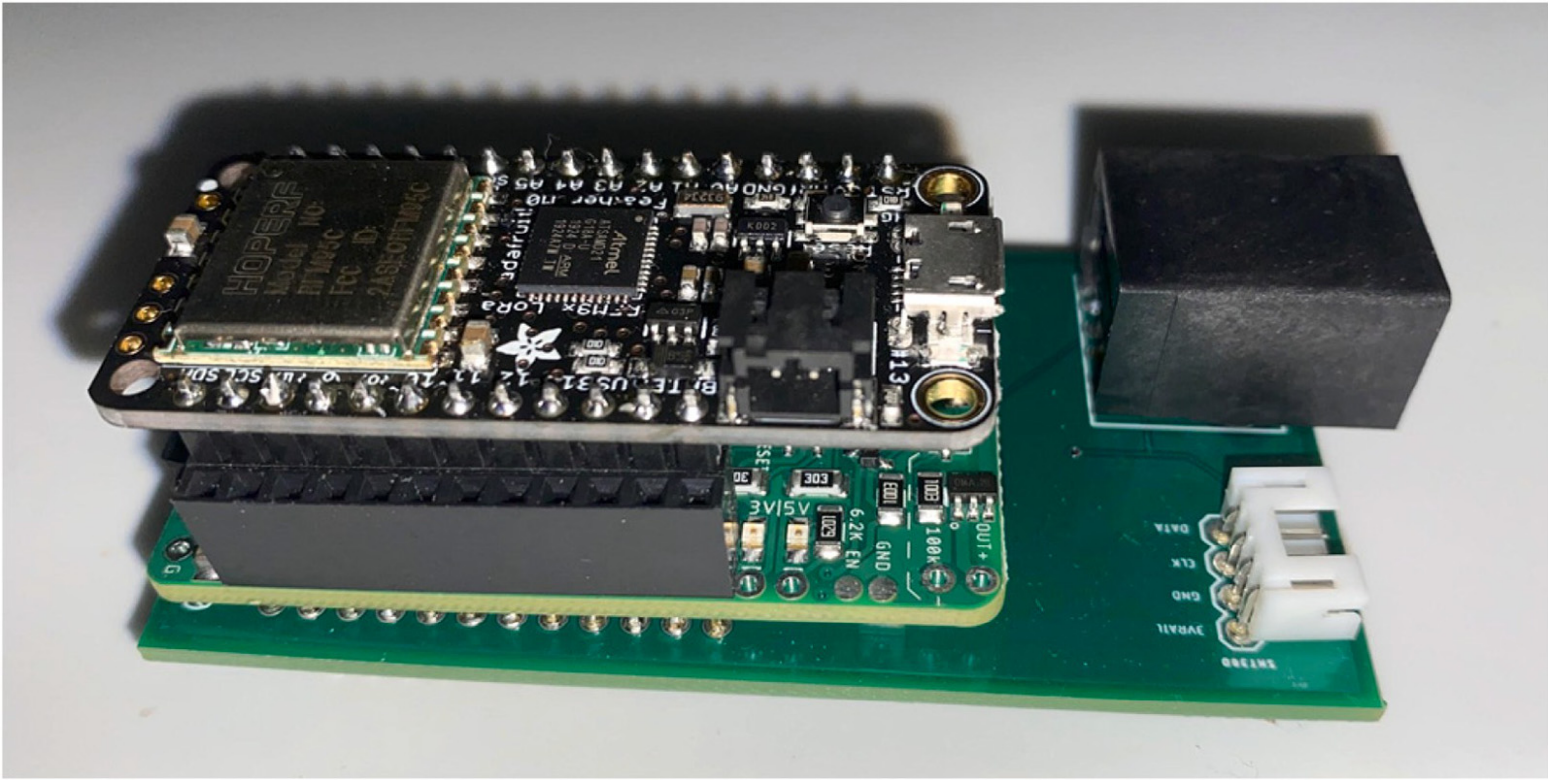
Stacked Feather/Hypnos on PCB.
4. Insert the Feather/Hypnos combination into the PCB ensuring the Hypnos board is between the Feather and PCB (Fig. 27).

Sensors: AS5311 and SHT30

1. Now obtain a 4ft. CAT5 cable to connect to the AS5311. Strip off the RJ45 connector on one side of the cable to expose the 8 internal wires (Fig. 28).

**Fig. 28:**
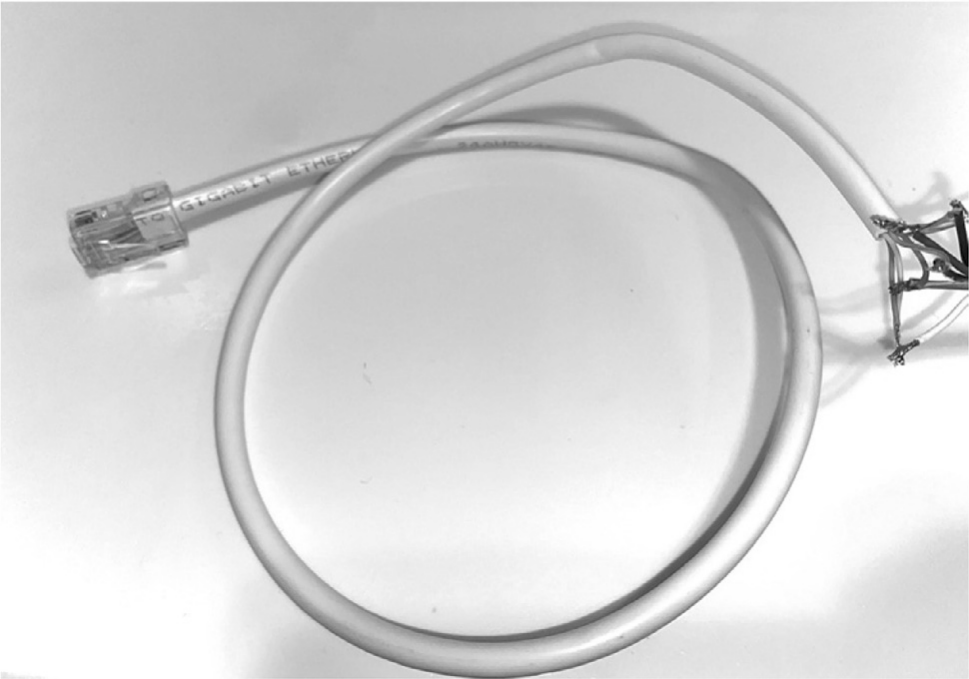
CAT5 cable with exposed end.
2. Feed the CAT5 through the pelican box starting from inside and feeding the stripped side out first (Fig. 29).

**Fig. 29:**
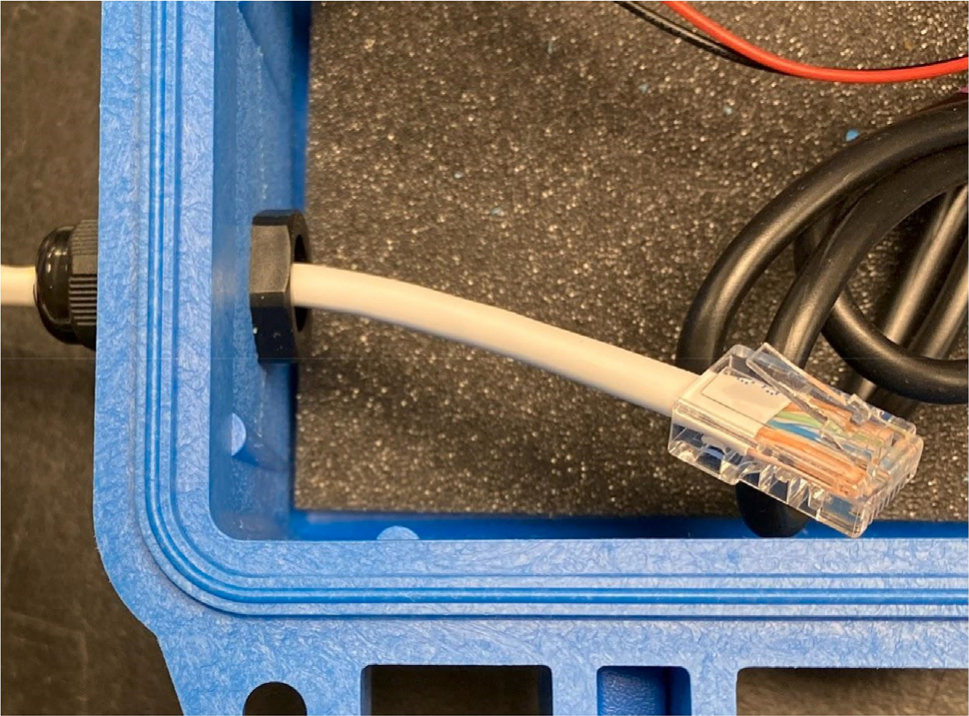
CAT5 cable with pelican box.
3. Inside the CAT5 cable, there are 4 twisted pairs (8 different wires). Only 6 of the wires are going to be soldered to the AS5311 with one pair remaining together for the 3Vrail (Fig. 30). Before soldering the 6 wires, cut the brown and brown/white wire down to the cable jacket as the sensor will not be using those wires. The rest of the wires are color coded and should be soldered to the ports (on the bottom of the board) on the AS5311 as follows (Table 1, Fig. 30):

**Fig. 30:**
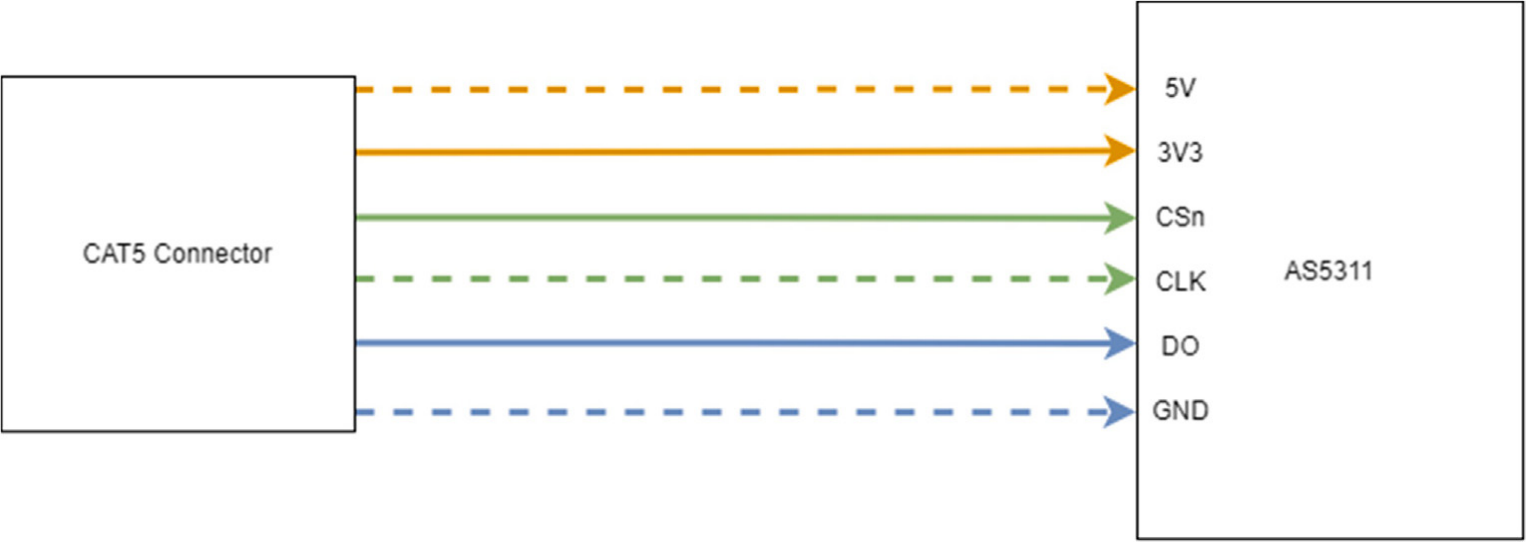
CAT5 to AS5311 Wiring Diagram.

**Table 1:** CAT5 Pinout.

Wire Color	AS5311 Pin
Orange / White	5 V
Orange	3 V3
Green	CSn
Green / White	CLK
Blue	DO
Blue / White	GND
Brown + Brown / White	Not in use

It is strongly recommended that hot glue be applied to the wires soldered onto the AS5311 to ensure the connections do not break.

### 5.5 JST connectors

Note: The button and SHT-30 Sensor should be on, or routed through, the Pelican Case before wires are connected.

1. There are 4 different connections on the PCB:
  a. 2 pin JST to Interrupt button:
    On the button secured to the Pelican case, solder the bare wire ends of the 2-pin female JST cable (Fig. 31). Orientation/polarity of the wire connection does not matter

**Fig. 31:**
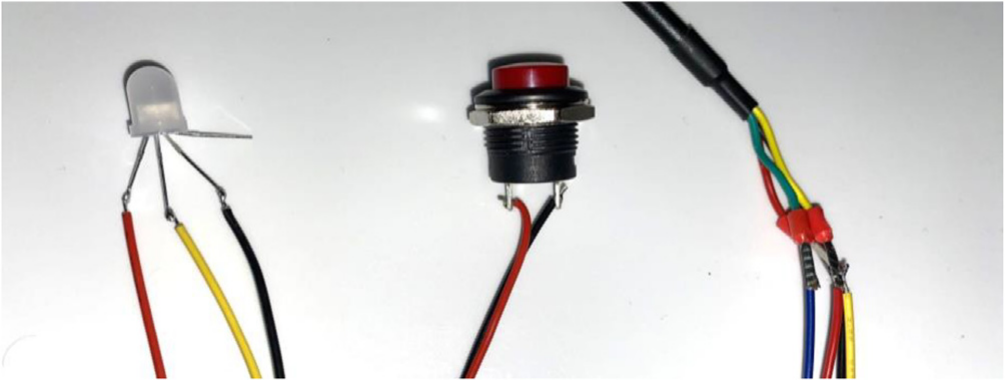
Wiring on the LED (left), button (center), and SHT-30 Sensor (right).
  b. 3 pin JST to Neopixel LED:
    Solder the bare wire ends of the 3-pin female JST cable to the Neopixel LED (Fig. 31). If necessary, use hot glue or heat shrink to surround the solder joint to make sure the connection is stable. Ensure that the connections are as follows:
    i. Red wire to Data In
    ii. Yellow wire to 3.3 V
    iii. Black wire to Ground
2. To tell what each pin is on the Neopixel, note the longest of the 4 pins. That pin is connected to ground. Orient the Neopixel so that the ground pin is the third pin from the left. Then, the pin order will be: Data In, 3.3 V, GND, and Data Out. Note that the longest pin on the Neopixel will not be used.
a. 4 pin JST to SHT30:
  Solder the bare wire ends of the 4-pin female JST cable to the ends of the SHT-30 Mesh-protected Weather-proof Temperature/Humidity Sensor (Fig. 31). Again, use heat shrink if necessary to cover the connection. According to the Adafruit webpage [19], the 4 wires from the temperature sensor are: Brown/Red = VCC (3-5VDC), Black = Ground, Yellow = Clock, Green/Blue = Data
3. Connections are as follows (Table 2):

**Table 2:** 4 pin JST to SHT30 wiring.

PCB Label	JST	SHT30 Color	SHT30 Pin
3VRAIL	Yellow	Red	VCC
GND	Black	Black	GND
CLK	Red	Yellow	CLK
DATA	Blue	Green / Blue	DATA
4. Plug all the female JST connectors and the CAT5 cable into the ports on the PCB (Fig. 32).

**Fig. 32:**
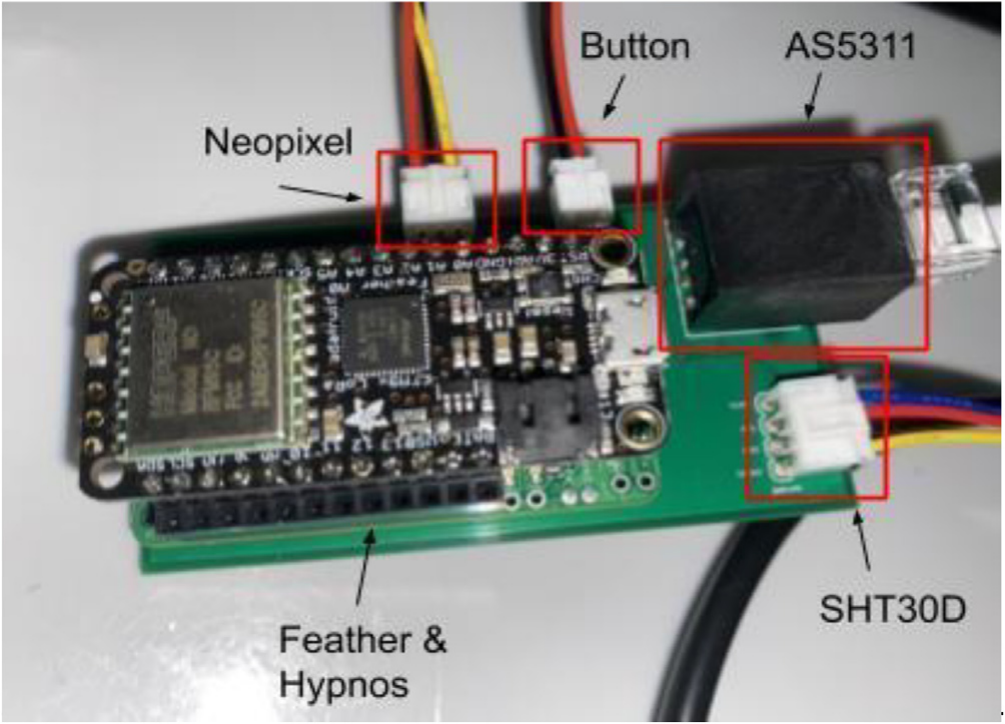
PCB with ports filled.

### 5.6 Final assembly

Put together the mechanical and electrical components using the Pelican Box and Sensor Plate.

1. Apply a layer of conformal coating to the face of the AS5311 board, however, do not put the conformal coating on the raised black sensor.
2. Take four 3–48 roundhead screws and remove some of the screw head using a file; reduce the head height to be about half the original.
  a. This is done to keep the screws from rubbing against the magnet and creating friction that would impede its movement, causing data inaccuracies.
3. Secure the AS5311 sensor onto the dendrometer.
a. Place the AS5311 sensor face up on top of the Long Body, and the Long Body on top of the Sensor Plate (orientation of Sensor Plate is unimportant). The front of the AS5311 sensor should be facing the T Magnet Mount (Fig. 18).
b. Slide the four filed 3–48 screws into the holes in the AS5311 from the top and around the Long Body and Sensor Plate.
c. Secure the four 3–48 screws with the brass 3–48 nuts on the underside of the Sensor Plate (Fig. 33). Tighten so that the Sensor Plate is firmly against the Long Body.

**Fig. 33:**
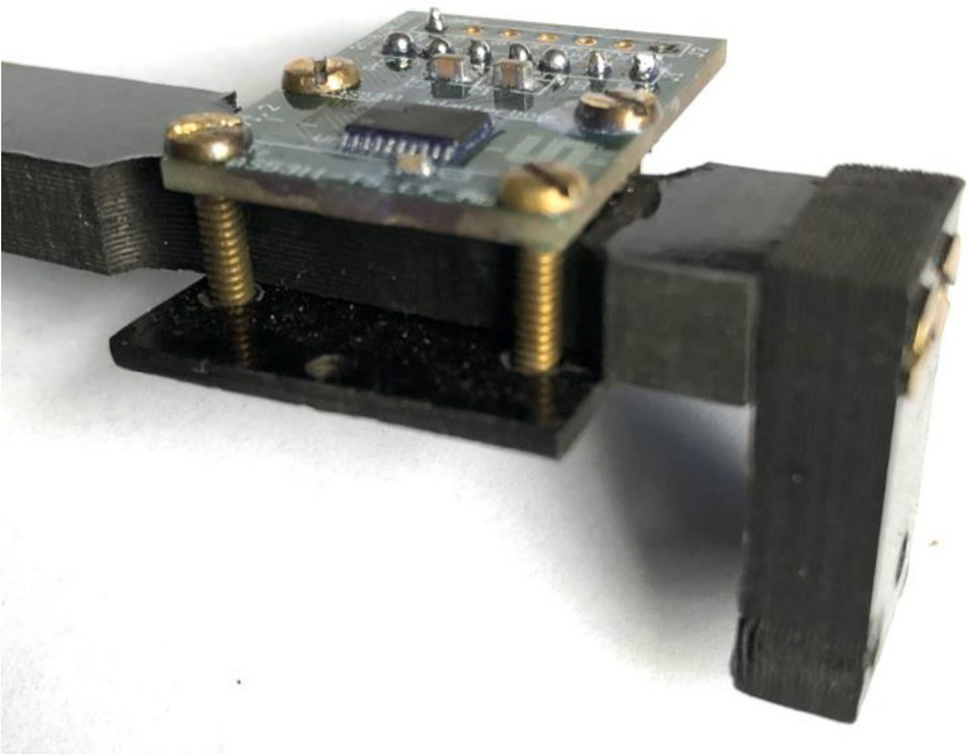
AS5311 sensor is held in place by the Sensor Plate (bottom of the screws). Sensor placement can be adjusted by loosening the nuts and sliding within the slots.
4. Loosen (but don’t remove) the thumb nuts on the Sandwich Grip so it can be slipped over the Rods and later tightened to clamp down and be held against a vine or stem (see operation guide).
5. Fit the LED bulb through the plug in the Pelican case and glue the base of the LED to the inside of the plug (the side that’s accessible from inside of the box) (Fig. 34). Apply glue or epoxy generously to the inside of the plug to keep the Pelican case sealed and weatherproof.

**Fig. 34:**
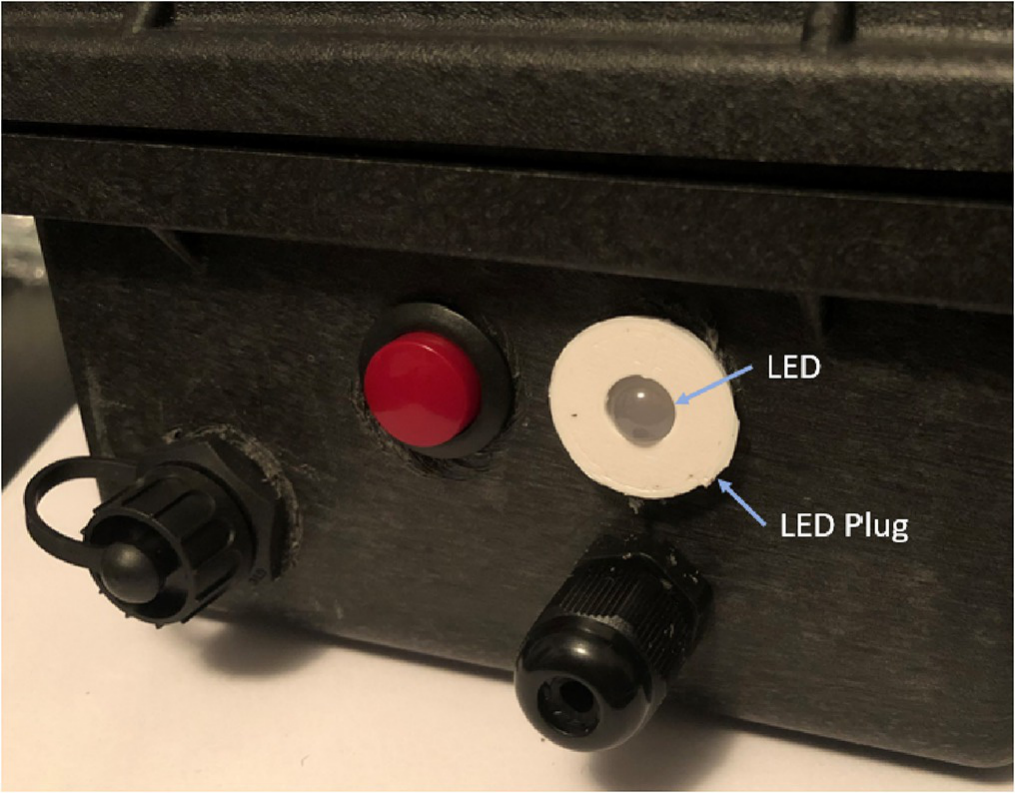
The LED in the LED plug, slightly protruding (this Pelican Case is also an example of another possible organization for the button, cable glands, and USB port than outlined earlier).
6. Connect the USB extension cable to the Feather M0 (Fig. 35)

**Fig. 35:**
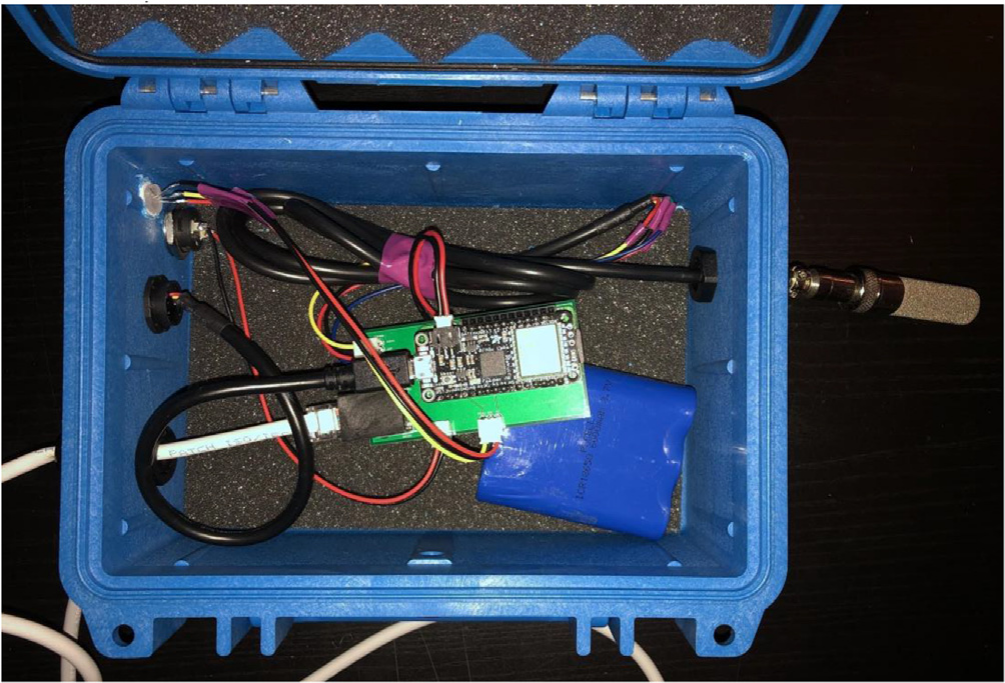
Pelican Case with all the electronics, connections, and parts put together inside.
a. The USB extension allows access via the port in the case to electronics and ability to update code.
7. To attach the Pelican Case Mount, some adjustments must be made to the case itself. Begin by removing the handle from the Pelican Case; this can be done by pushing out the pins holding it in place. On the Pelican Case Mount Top and Pelican Case Mount Bottom, tap the labeled holes (Fig. 36) to ¼”-20. Both mounting pieces are a friction fit design. Align the mounts with the ridges along the back and sides of the case, ensuring that they are fully seated against the outer wall. Using the ¼-20 bolts, secure the mount to the case through the holes where the handle used to be pinned [22].

**Fig. 36:**
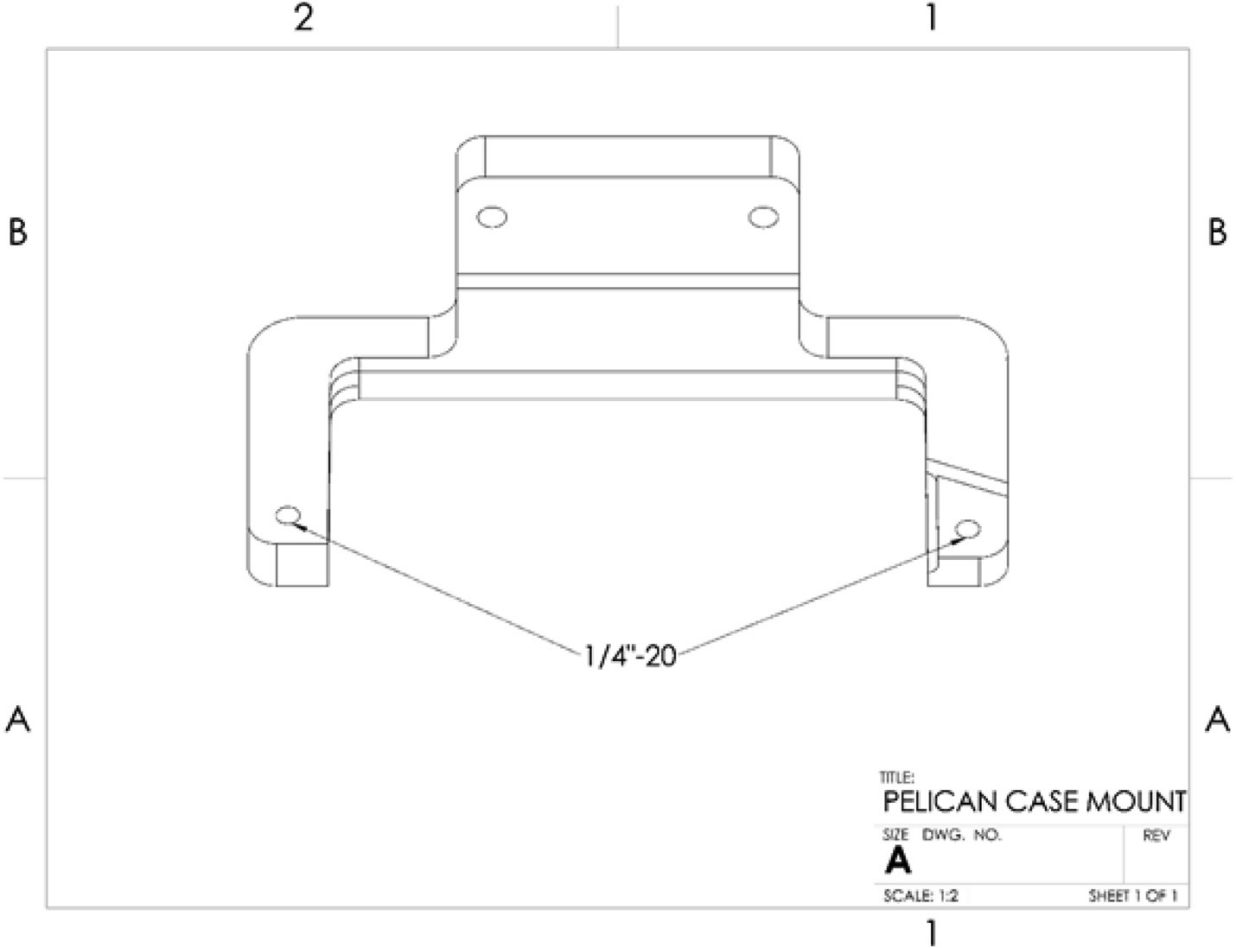
CAD drawing showing location of tapped holes on Pelican Case Mount Top and Bottom [22].

**Fig. 37:**
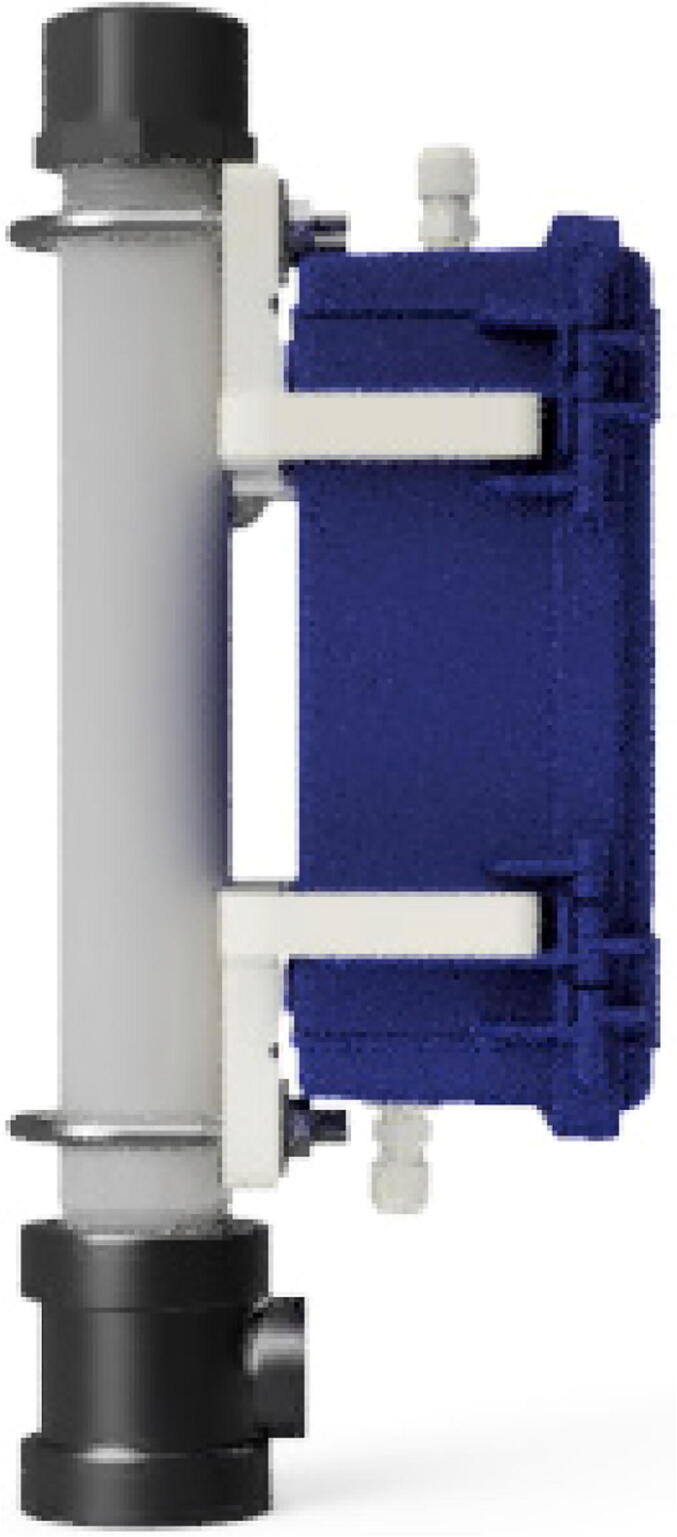
Pelican Case Mounted to a PVC pipe using the Pelican Case Mount Top/Bottom (the actual PVC pipe used will be much longer than the one shown).

## 6 Operation instructions

To use the dendrometer, software must be flashed to the Feather M0 board; Section 6.1 will detail this process and provide guidance on accessing and understanding the data. After the software is uploaded, the dendrometer is ready for field operation and can be installed on a plant stem or vine (Section 6.2). An LED indication system is included in the device to validate the dendrometer is operating correctly; instructions on using this verification tool are provided in Section 6.3.

### 6.1 Electronics / computer setup

#### 6.2.1 Getting dendrometer source code

The software that this prototype relies on is all stored at the OPEnS Lab Github, which can be found at the Dendrometer Zenodo repository. Follow the link and instructions below to get the software onto your computer.

In order to get the code onto your local computer, follow these steps:

1) From Github, click on the ‘Code’ button and download the ZIP file. From Zenodo, scroll down to Files and download the ZIP file.
2) Extract the files from the ZIP folder to a folder you can find later.

#### 6.2.2 Setting up Arduino editor (preparing to upload software)

In order to upload the code onto the Feather M0 board, the Arduino IDE software, as well as all of the dendrometer’s dependencies (external code that it relies on), need to be downloaded onto the local computer.

Follow the steps provided in the Arduino and Loom Manual Setup guide in order to ready the IDE for uploading the software. There are two board profiles that are used: the Arduino SAMD and Adafruit SAMD board. Install the most up to date profile for the Arduino SAMD, but the Adafruit board needs to be installed with version 1.5.7. This is because of an issue with the SD starting up with a more recent version. The Loom version to install is 2.5.

#### 6.2.3 Uploading the code

Now that the computer has the Arduino IDE, the dendrometer software can be uploaded to the Feather M0.

**Fig. 38:**
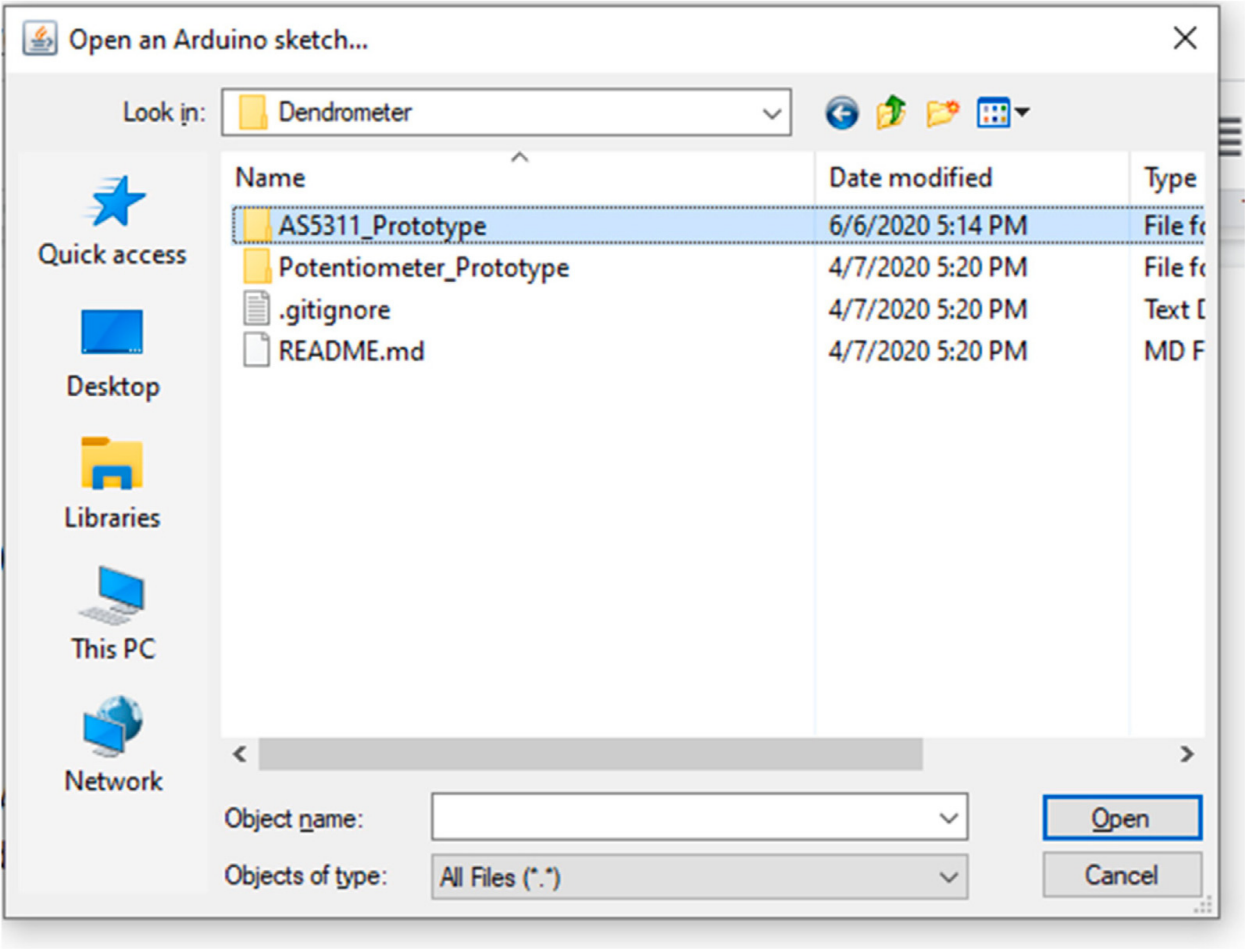
File structure for dendrometer code.

Open the Arduino IDE. From there, click ‘File’ then ‘Open’ and navigate to the location where the source code was downloaded to in ‘Getting Dendrometer Source Code’ section (Fig. 38).

**Fig. 39:**
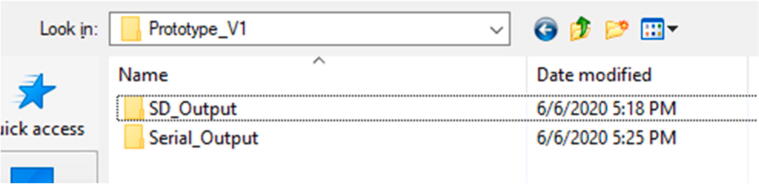
Contents of Prototype_V1 Folder.

Enter the AS5311_Prototype > Prototype_V1 folder and there are two choices (Fig. 39).

The SD_Output folder will contain the source code for measuring and outputting data to an SD card. The Serial_Output folder will contain the code that will measure and output data straight to the Serial Monitor on the computer screen (but will not save the data anywhere).

Double click on whichever folder you would like to use, and double click the file that matches the folder name that is presented inside the file (either SD_Output or Serial_Output) and the code should appear on the IDE. Then follow these steps:

1. Ensure that there is a μSD card inserted into the Hypnos board if the SD code is chosen (Fig. 40). The coin cell battery should also be inserted in the socket on the bottom of the Hypnos board so the RTC can continue to run during the sampling period.

**Fig. 40:**
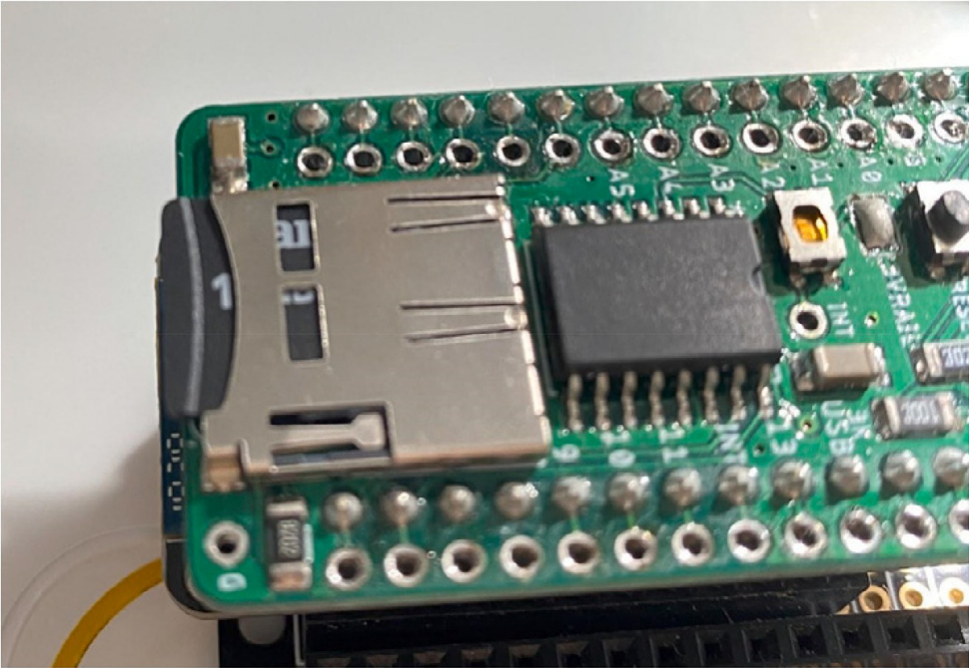
μSD card in Hypnos.
2. After connecting a data enabled micro-usb cable to your computer and the M0 board, ensure the PORT with the “Adafruit Feather M0” label is selected (shown in Fig. 41) and upload the code.
  a. SD_Output will take 5–10 min to upload. The Serial_Output code will take under a minute. It is a good idea to start out with Serial_Output first to get an idea of how the program works before moving to SD.

**Fig. 41:**
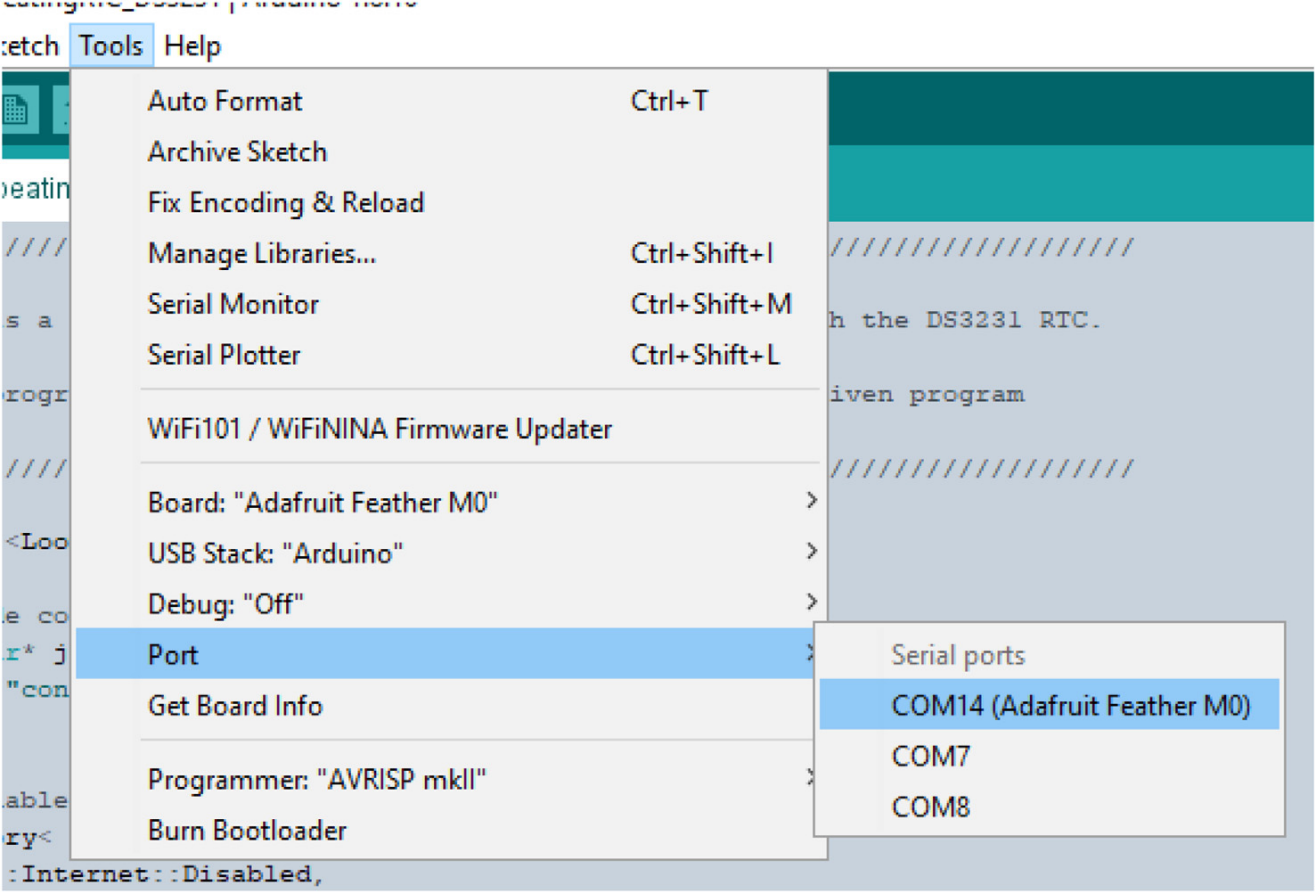
Port Selection.
i. If getting an error message on the IDE, hit the reset button on the Hypnos board (Fig. 42) immediately after clicking ‘upload’ on the IDE.

**Fig. 42:**
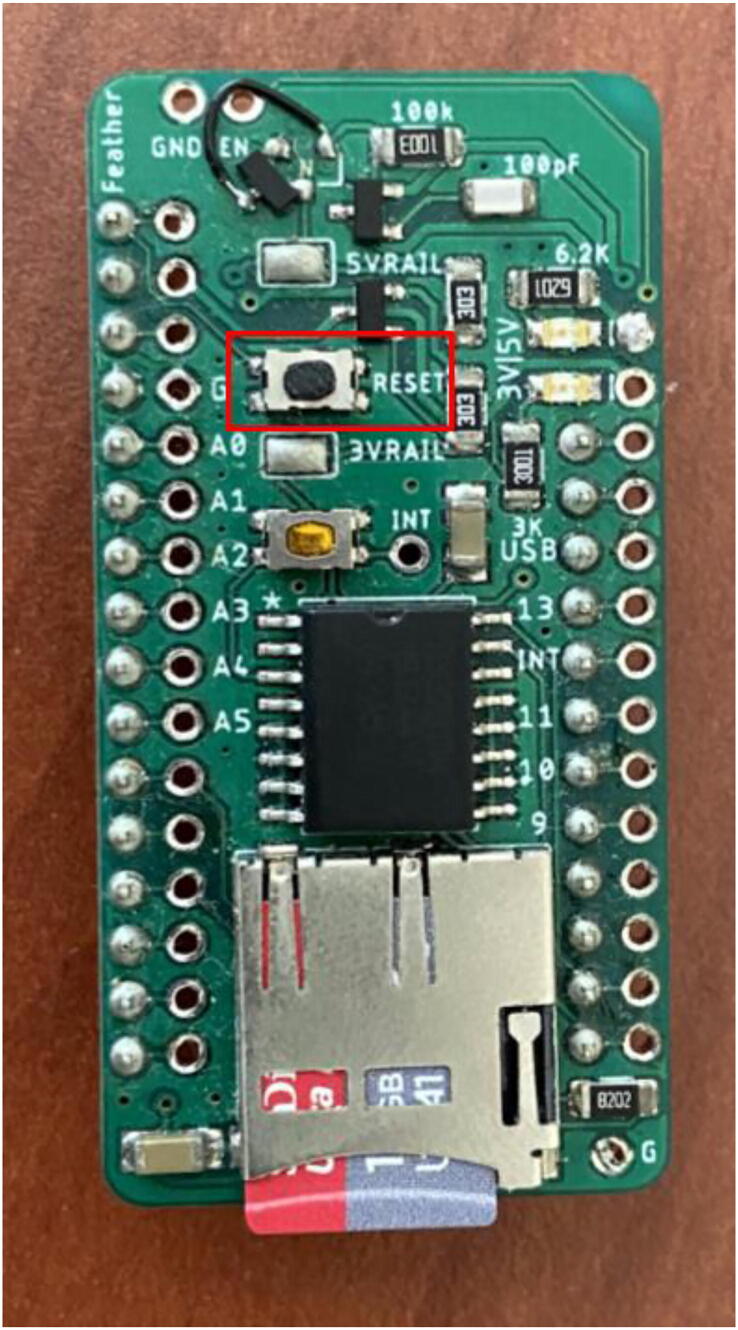
Hypnos Reset Button.
3. If there are no error messages and you have been alerted that the upload is complete, a message like that shown in Fig. 43 should appear.

**Fig. 43:**
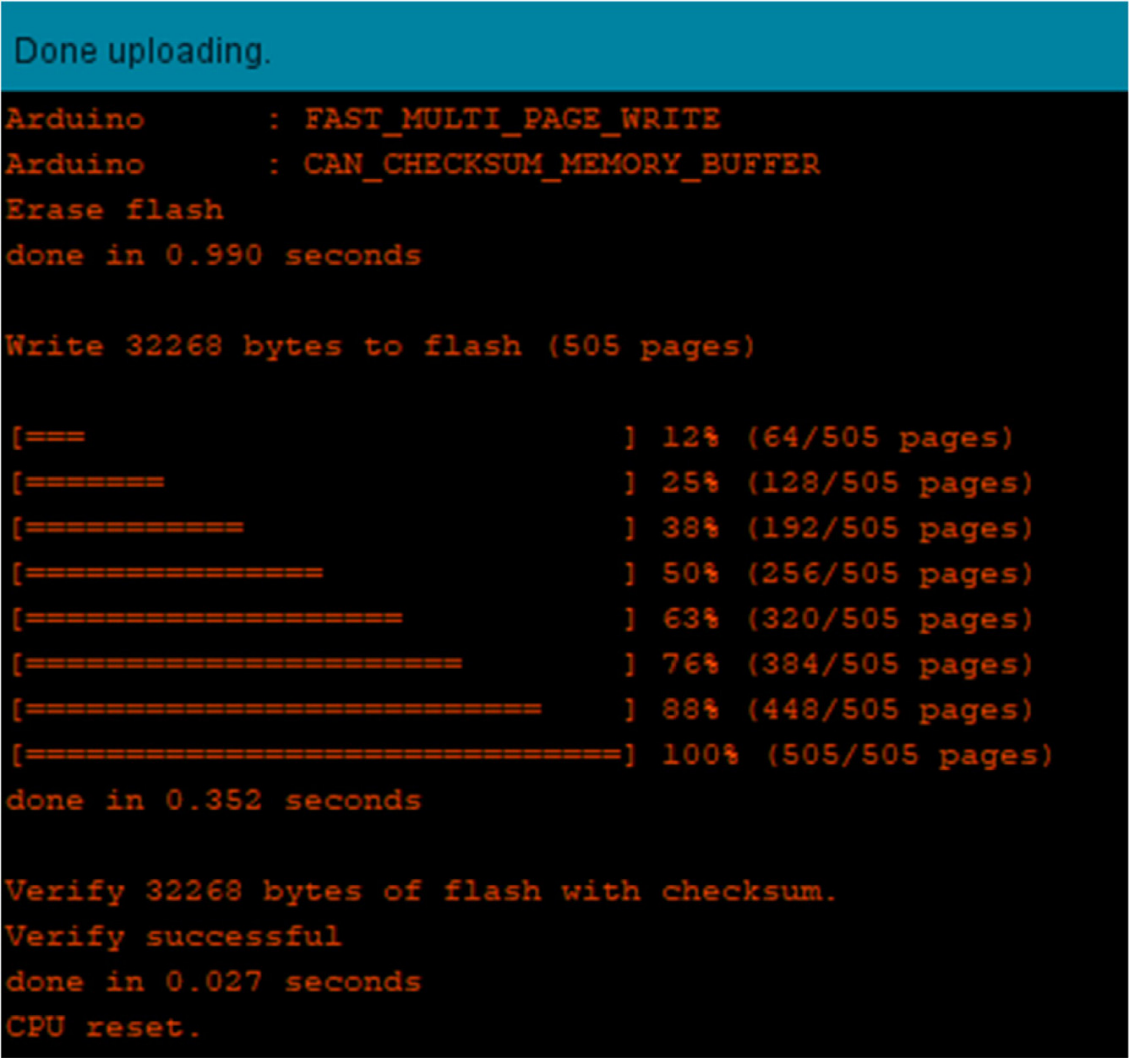
Successful upload output screen on the Arduino IDE.
a. After a successful upload, the code will remain on your board and the board may be disconnected from the computer.

#### 6.2.4 Using the dendrometer prototype

With the prototype built and the software flashed into the board’s memory, the prototype is ready to be used. The board can be powered from the computer via micro-USB into the power jack on the Feather M0 or a 3.7 V LiPo battery in the JST connector on the Feather (see Bill of Materials for recommended battery).

1) Using the dendrometer (for both SD and Serial code)

Once the system is plugged into a power source, it searches for the magnet and its position. The program will keep searching for the magnet and for it to be in a position that will read accurately before it continues.

**Fig. 44:**
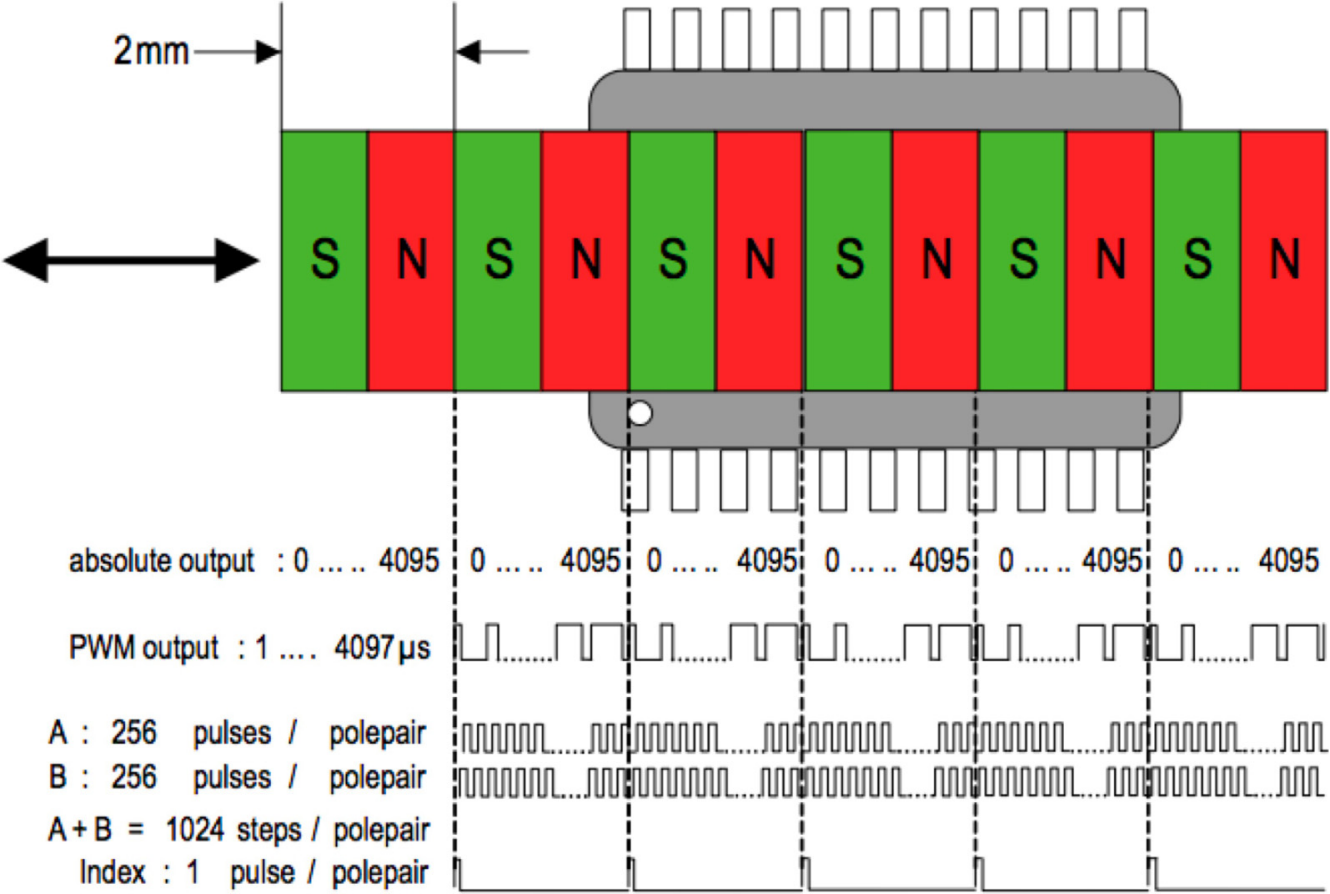
Diagram of magnet and AS5311 sensor [14].

The dendrometer senses displacement by comparing all future measurements to the initial measurement that the board takes ***upon setup*** as well as the ***immediate previous measurement****.* This means once the prototype is turned on and the magnet is positioned properly, a measurement is taken and is used as a reference to calculate the displacement of the next measurement, which will be used to calculate the next, and so on and so forth. This relative measurement referencing is required because the sensor can only differentiate between locations, output as a value between 0 and 4095, within a single pole pair of the magnet at a time (Fig. 44). The specified magnet has a pole pair length of 2 mm, meaning each north and south pole pair has a combined width of 2 mm. It is therefore crucial that the magnet **does not move more than 2 mm** between measurements as the sensor will not be able to correctly measure that displacement.

2) Recognizing that the dendrometer is working

**Fig. 45:**
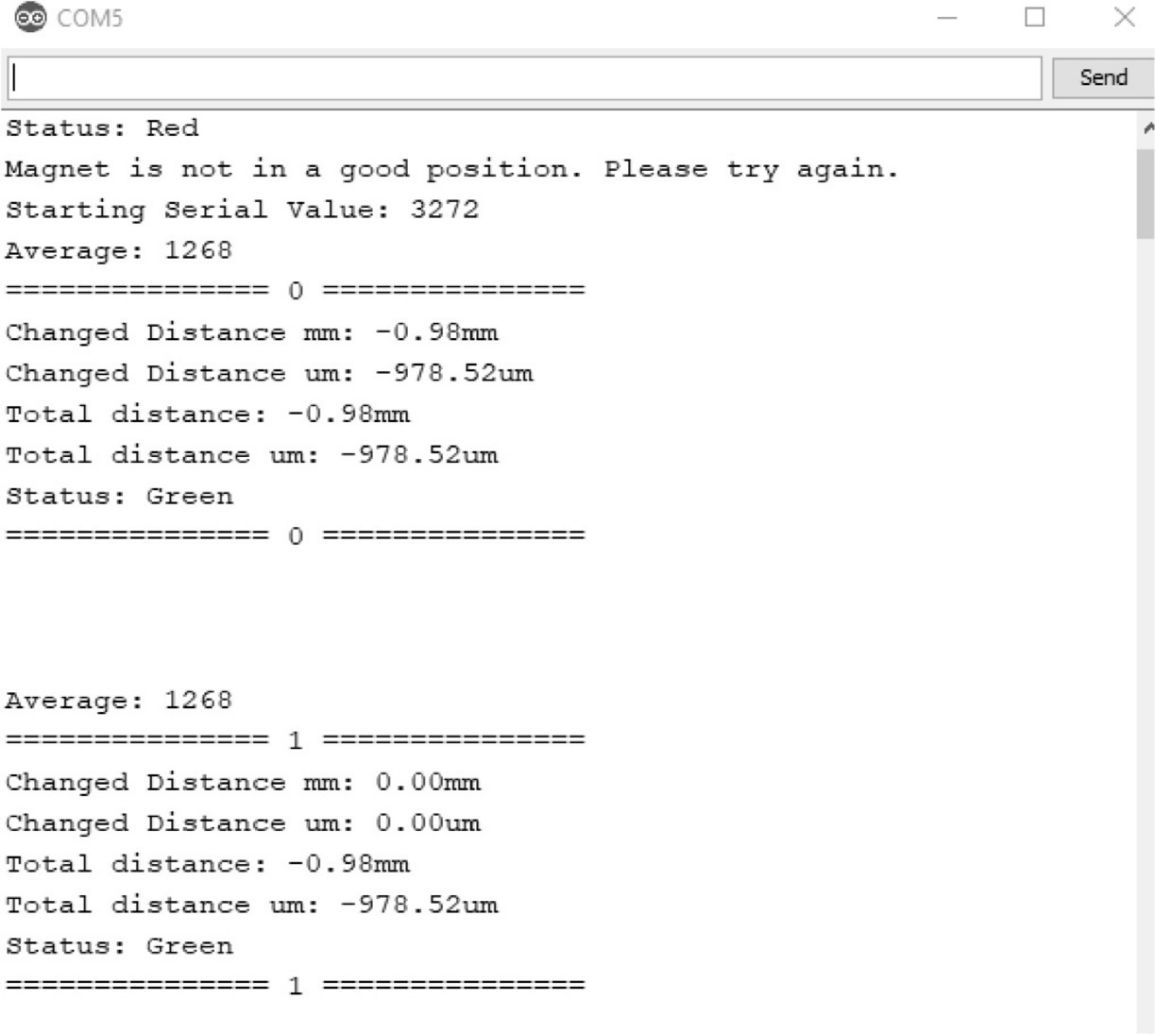
Serial Monitor of Serial_Output Program.

For the **Serial_Output** program, ensure that the dendrometer is connected to a computer with Arduino downloaded and the correct port is selected. After clicking on the serial monitor (on the top right of your Arduino IDE), you should see the following screen shown in Fig. 45.

In the serial monitor, ‘Total distance’ shows how much the magnet has moved relative to when the dendrometer powered up. ‘Changed distance’ shows the difference between the current total distance and the immediately previous total distance.

Hitting the reset button on the Hypnos board will reset the program and will start the output at index “=====0=====”.

If the displacement seems abnormal, such as the displacement suddenly makes a jump back and forth despite the magnet only going one direction or it jumps positive to negative, the magnet has moved too far in between two measurements. If this is the case, the most probable cause is that the magnet has moved more than 2 mm during measurement. Ensure the setup doesn’t move more than 2 mm between each measurement.

**Fig. 46:**
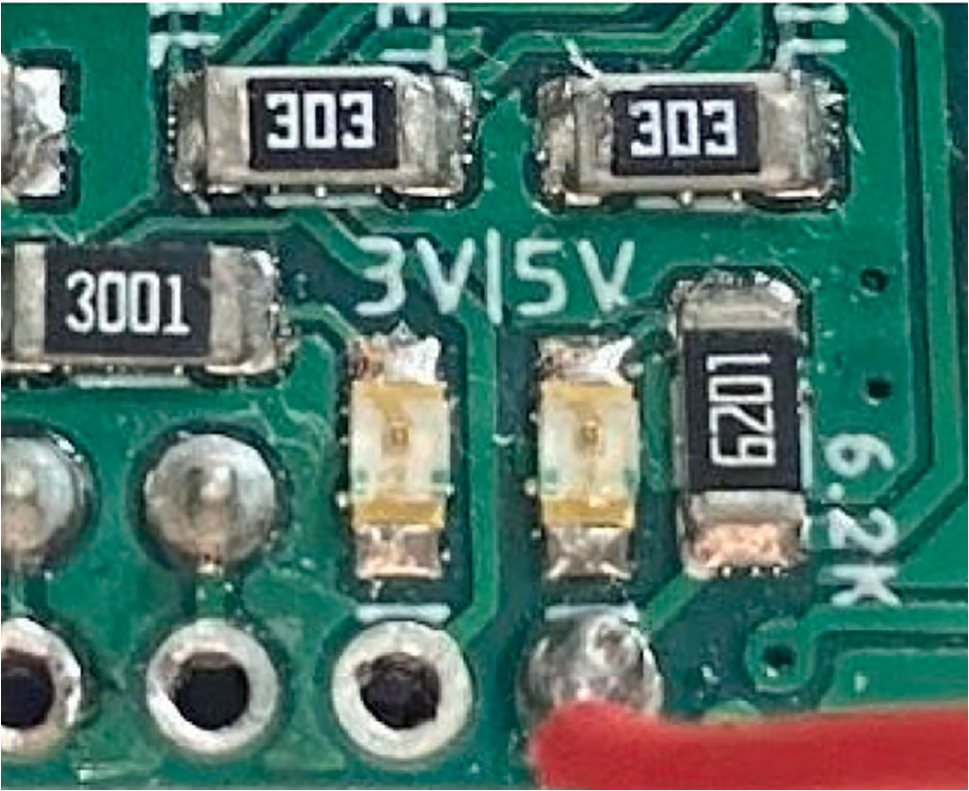
Hypnos LEDs.

For the **SD_Output** file, the two LEDs on the Hypnos board (Fig. 46) are an important indicator of what is occurring.

**Fig. 47:**
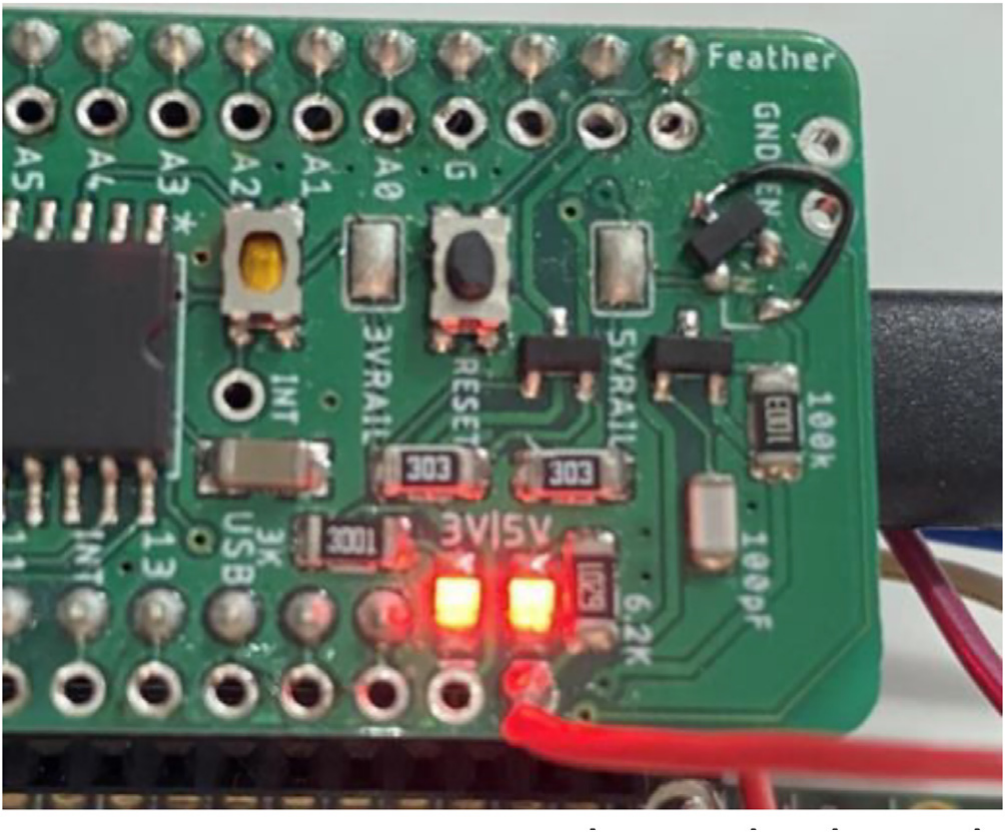
Hypnos is awake and taking data.

**Fig. 48:**
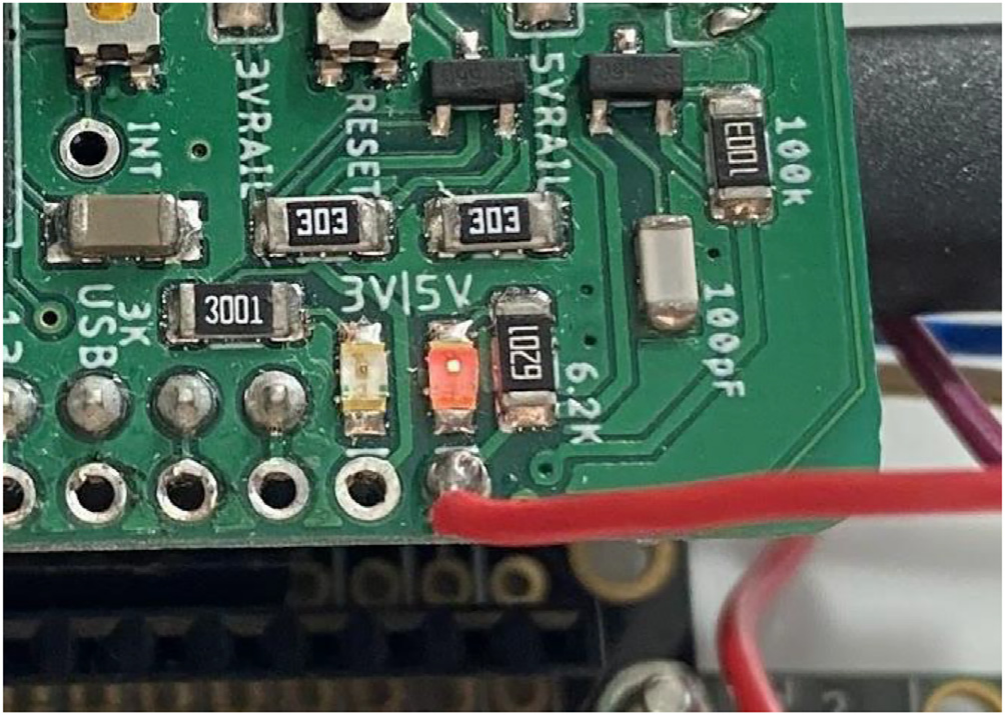
Hypnos is asleep and not taking measurements (LED brightness may vary depending on board).

When the Feather is active and awake, these LEDs will shine brightly, indicating that everything is configured (Fig. 47) which is most prevalent during startup. Make sure that during this time, the magnet is above the sensor and is not moving. After a couple seconds, it should return to being dim (Fig. 48), meaning the dendrometer is asleep and the main program execution has begun.

During the sleep period (when the LEDs are dim), the dendrometer is in low power mode and will not be reading or logging data. The magnet can move during this period, as long as the movements are less than 2 mm-- otherwise it can corrupt the data that will be read once it turns on again.

After the first initialization step (bright LEDs, Hypnos is awake), all of the following awake periods should be around 1 s long due to the data collecting and recording which can not be changed. During this period, the program is taking multiple measurements of the magnet and averaging them to be written to SD.

The dendrometer is pre-set to wake up at a period of every 15 min, but this can be changed by the user. This will be described in Section 6.1.5.

**Fig. 49:**
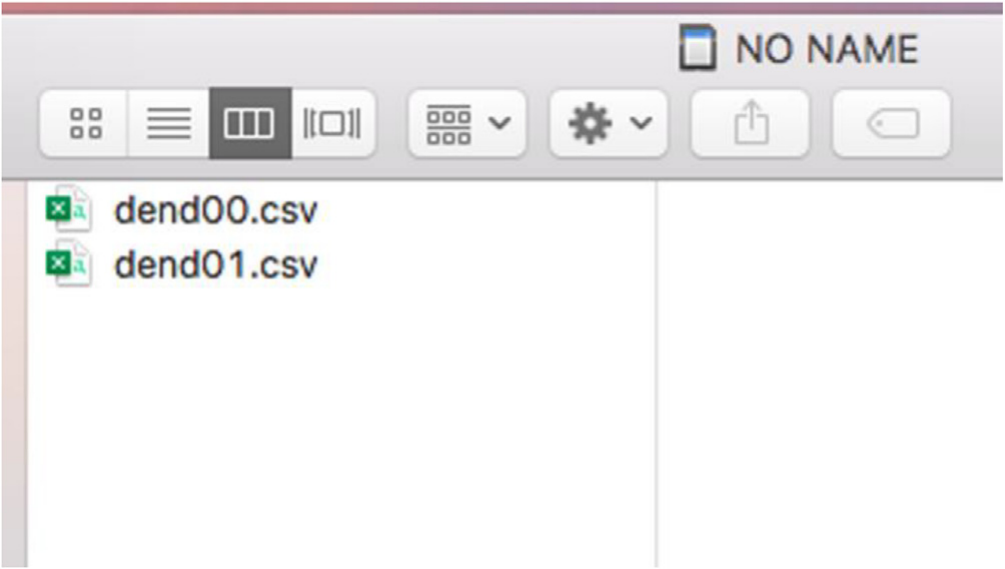
Two .csv files generated from the program, dend00.csv and dend01.csv.

**Fig. 50:**
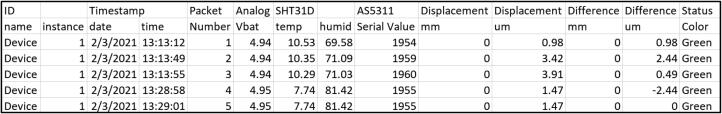
How the .csv files look in a spreadsheet.

All of these readings will be outputted to a dend##.csv file (Fig. 50) where the ‘##’ will automatically increment with each power up or reset. The file structure on the microSD card should look similar to Fig. 49.

There are various columns that represent the date and time, battery level (Vbat), temperature and humidity, and the AS5311 serial and position values as well as the magnet status. The magnet status will be red, yellow, or green with red being the magnet was in a bad position to green being a good position. The ‘Difference’ column will tell the relative change from the last measurement point and can be used to check if something went wrong.

#### 6.2.5 Modifying the sampling period

**Fig. 51:**
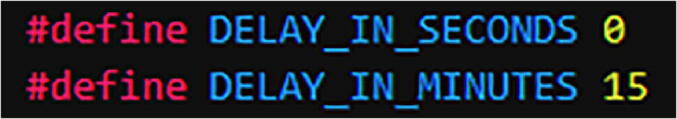
Testing Period Variables.

At the top of both the Serial_Output.ino and SD_Output.ino files, there are definition statements that assign the testing period (Fig. 51).

This specifies how often measurements are being taken. By default, this is set to 15 min and 0 s meaning every 15 min the position of the magnet is being read and outputted to wherever it is specified depending on the program. It is recommended to use the default testing period for testing grapevines.

If a new sampling period is desired, the user must change these values in the code and re-upload to the dendrometer board through the Arduino IDE. To check if the sampling period changed, record the time in between the Hypnos LED flashes to verify that the period changed. For the Serial mode, the serial monitor should now be outputting new values every (x) minutes / seconds that was specified. Similarly, for the SD mode, the 2 LEDs on the Hypnos board should turn on every (x) minutes / seconds.

### 6.3 Installing the dendrometer on a vine

Now that the electronics are functioning, the Pelican Case is fully set up, and the mechanical assembly is complete, the dendrometer is ready to be deployed on a vine or plant stem.

1. Choose a plant to install the dendrometer on. It should have a vine or stem with a diameter of 23–40 mm, ideally 0.3–1.0 m off the ground for Pelican Case mounting purposes.
a. Ensure there is at least an inch of relatively flat/smooth vine to mount the dendrometer to.
b. Remove leaves or any other possible obstructions nearby the mounting site.
c. The more vertical the orientation of the vine or stem, the better.
2. Remove the bark from where the dendrometer will be taking measurements by peeling and rubbing until most of it is gone from that area.
3. Drive the PVC pipe into the ground so that it is stable but at least 0.75 m remains above ground for mounting the Pelican Case.
a. You will likely want the PVC pipe in between the trellis or T posts that the grapevines are held up by.
4. Use the U-bolts to attach the Pelican case to the PVC pipe. See Fig. 37 for reference.
5. To secure the dendrometer to the vine, the hose clamp will be used. If on, remove the Sandwich Grip from the Rods. Orient the device so that the magnet is above the sensor (Fig. 52).

**Fig. 52:**
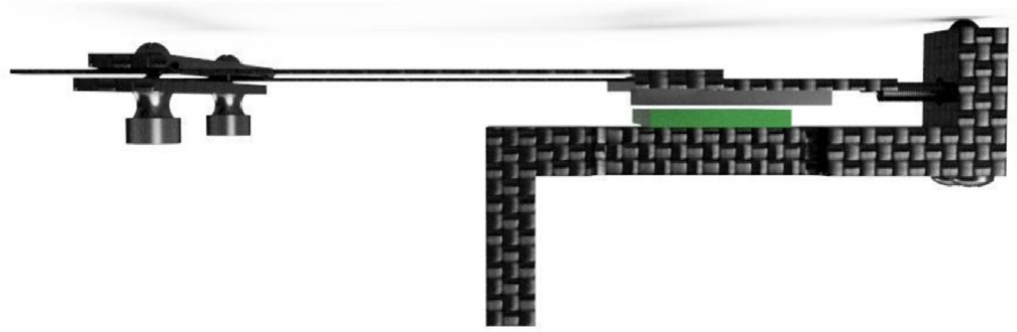
Dendrometer orientation without the vine. Stem or vine will be in between the Sandwich Grip and Vine Magnet Mount when installed.
6. Holding the dendrometer against the vine, tighten the hose clamp around the Vine Contact Mount and vine until it is snug and does not wiggle (Fig. 53).

**Fig. 53:**
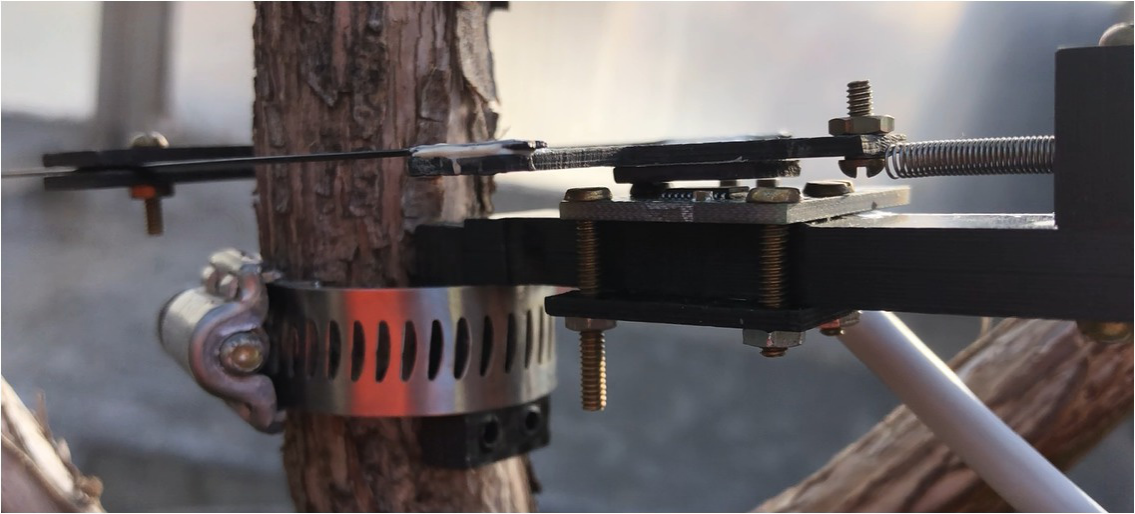
A hose clamp holds the dendrometer on a stem or vine by wrapping around the Vine Contact Mount and tightening against the vine.
7. With the Rods on either side of the vine, take the Sandwich Grip and slide it along the Rods while lightly pulling the spring (so contraction is possible and accounted for if it occurs before the plant expands or grows more). Once the edge of the Sandwich Grip is firmly against the vine and is perpendicular to the Rods, use the thumb nuts to clamp the Sandwich Grip on the Rods.
a. The goal is to align the edges of the AS5311 board (green) and the magnet furthest from the Spring Hold. The magnet and magnetic position sensor on the AS5311 should be parallel (Fig. 54).

**Fig. 54:**
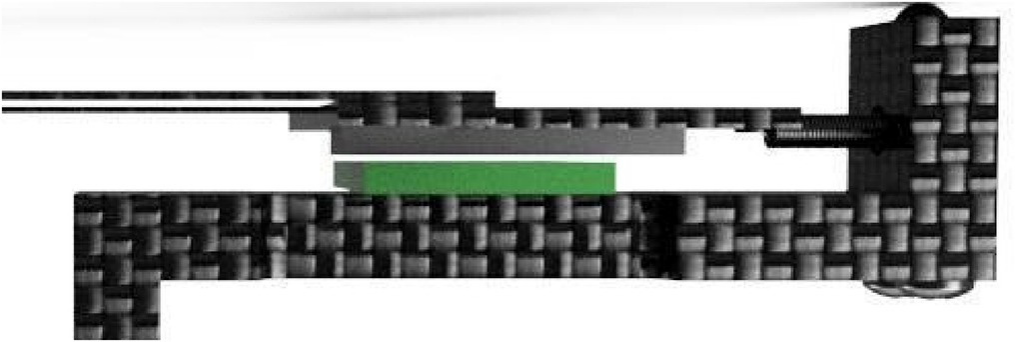
Close up of magnet (gray) and AS5311 sensor (green) initial alignment. (For interpretation of the references to color in this figure legend, the reader is referred to the web version of this article.)
8. Open the Pelican Case and plug the battery into the Feather M0.
9. To adjust the orientation of the sensor and the magnet so that the alignment is sufficient for the AS5311 sensor to make accurate measurements, the LED indication system will be employed. The distance between the sensor and the magnet must be 0.2–0.4 mm according to the AS5311 datasheet [14]; the Spring Hold is designed so that if the T Magnet Mount is exactly parallel to the Long Body, this distance will be achieved. However, given the precision required, this can still be difficult; the LED indication system can be used to make slight adjustments of the magnet and Sandwich Grip positions. The dendrometer will not begin recording data until the correct distance is achieved, in which case the LED will light up green (note that the color change might take a few seconds to appear). Adjust until the LED is green, then leave it in that position (the button does not need to be used upon initial startup of the dendrometer). This completes the process of setting up the dendrometer (Fig. 55).

**Fig. 55:**
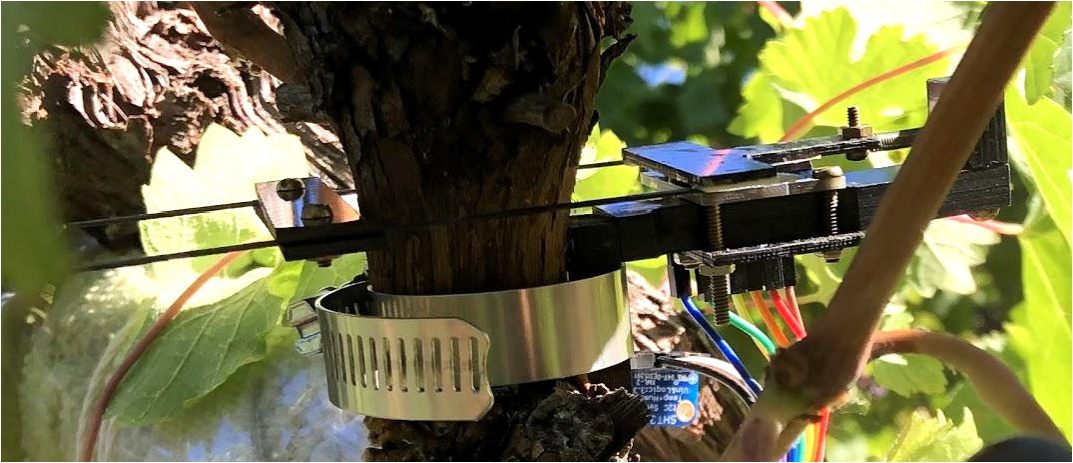
Dendrometer successfully installed on a grapevine (note the wiring is different from build guide).
a. This is the most finicky part of the set up and will likely require some patience and possibly several small adjustments/tests. Placing a 0.2–0.35 mm feeler gauge between the magnet and AS5311 sensor might be helpful.

### 6.4 LED indication system

The LED Indication System is implemented to easily check that the dendrometer is actively collecting data. When the button is pushed, the device will check the distance between the magnet and sensor to see if it is still within the required range of 0.2–0.4 mm. If it is, the LED will turn ***green*** for a few seconds; this indicates that the dendrometer is still accurately recording data. If the LED turns ***red***, something may have caused the magnet to shift relative to the sensor, in which case the data may no longer be valid during the previous testing period (when looking at the data, you will likely be able to see a jump when the misalignment event occurred). If this happens, use the LED indication system to adjust the magnet and Sandwich Grip until the LED is green when the button is pushed. Alternatively, open the Pelican Case and hit the ‘Reset’ button on the Hypnos board, then refer back to step 7 of the setup process to get everything up and running again. This approach will create a new data file on the SD for the coming measurements. In rare cases, the LED will appear ***yellow***; this means that the alignment is still in range, but is on the very edge. This could impact the precision of the measurements; it is recommended that the same procedures for adjustment be followed as when the LED is red. However, if yellow, the data trends can be expected to still be valid, although slightly less accurate.

## 7 Validation and characterization

The dendrometer has been validated in three contexts: in lab for temperature-independence of the displacement sensor (Section 7.1); in the field for vineyard operation (Section 7.2); and in the field for data trend accuracy (Section 7.3). The three experiments highlighted below demonstrate the successful operation of the dendrometer, as well as indicate device capabilities and limitations (Section 7.4).

### 7.1 AS5311 sensor temperature dependence test

To ensure that the linear magnetic sensor readings are not affected by ambient temperature fluctuations, an oven test was performed with the sensor. The AS5311 and magnet were aligned according to the specifications on the AS5311 data sheet, placed in a small oven, and remained static throughout the test; the oven was used to create temperature changes. Temperature was measured using an SHT31-D and data was recorded to an SD card. Measurements were recorded every five seconds.

**Fig. 56:**
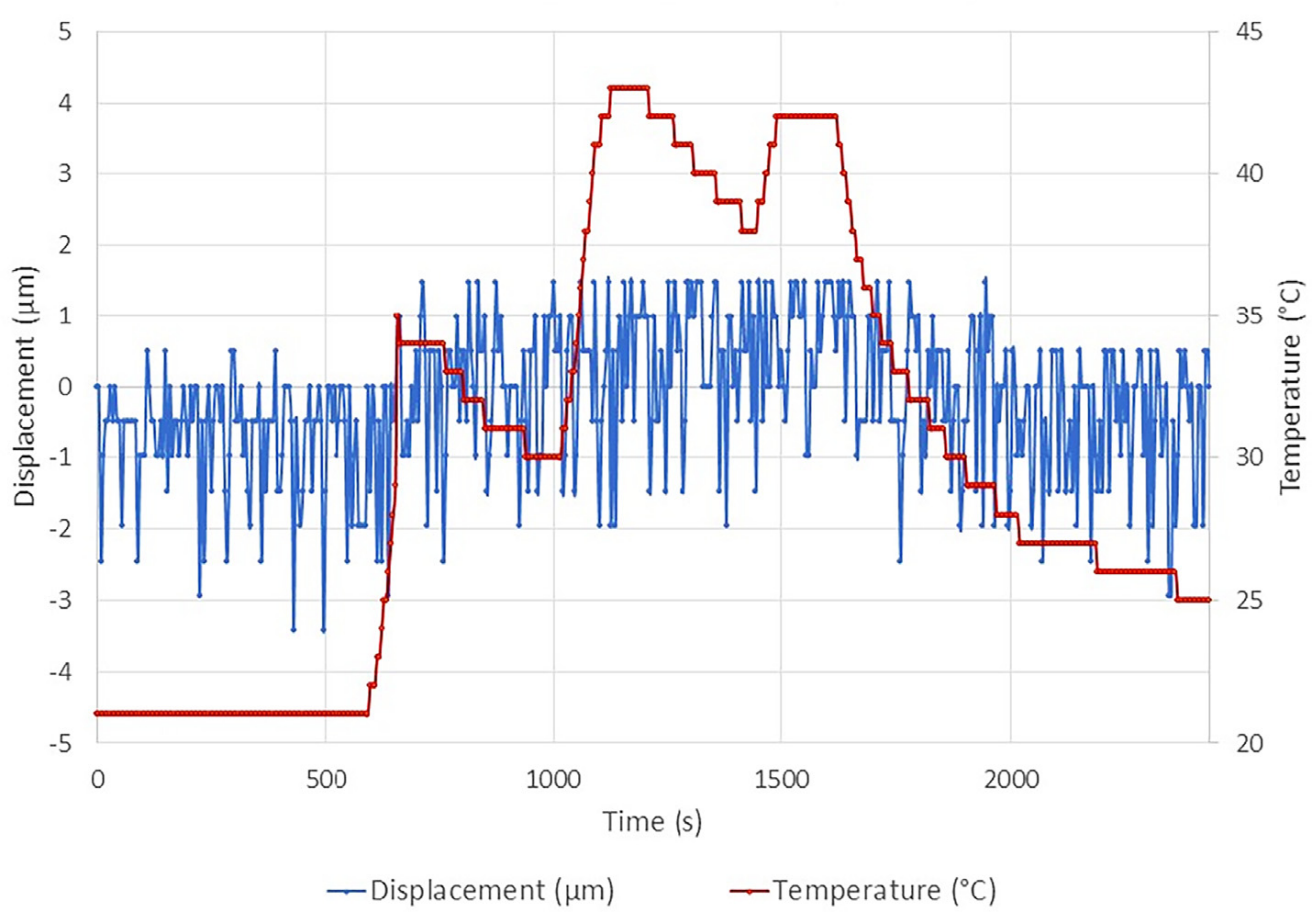
Lab test results for temperature dependency of AS5311 sensor measurements. Temperatures ranging from 10 to 42 °C were produced to evaluate if the sensor output readings varied.

The results demonstrate that this magnetic sensor has an error of 0.002% within this temperature range (Fig. 56). As can be seen in Fig. 56, with fluctuating temperatures, the magnetic sensor is able to output a consistent value; displacement measurements go from a minimum −3.43 µm to a maximum 1.47 µm, creating an overall change of 4.9 µm. The magnitude of the noise in displacement is essentially negligible since daily stem fluctuations are expected to be in the range of 100–200 µm.

### 7.2 Vineyard deployment

**Fig. 57:**
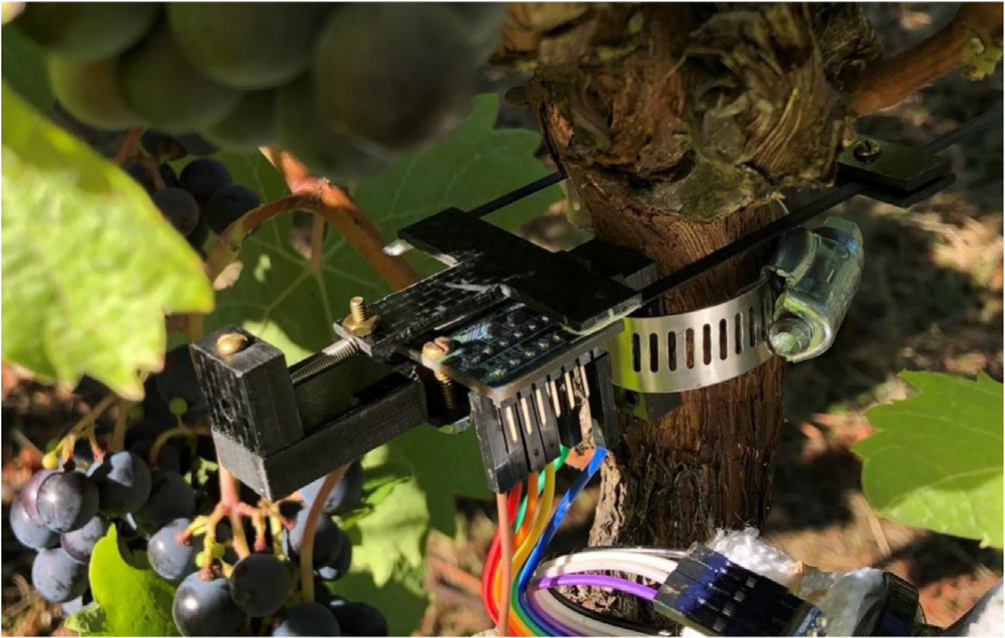
Dendrometer prototype installed on grapevine at OSU Woodhall III Vineyards. Note there are small differences in the wiring setup and fasteners from those described here.

Note: Gaps in the data exist from removed data values. At times, the dendrometer recorded a serial position that did not reflect true position, which was evident because the data would jump to the beginning or end of the serial values (either 0 or 4095). To reduce noise in the graph, these values were removed.

The primary long-term deployment took place for a week at OSU's Woodhall III Vineyard near Alpine, OR in October 2020. One dendrometer was installed on a vine (about ten years old) with a diameter of 26 mm (Fig. 57). A second dendrometer was placed on a Pyrex graduated cylinder (known to have an extremely low coefficient of thermal expansion of 4 µm/(m°C) [16]) to evaluate potential temperature dependency present in the dendrometer system.

**Fig. 58:**
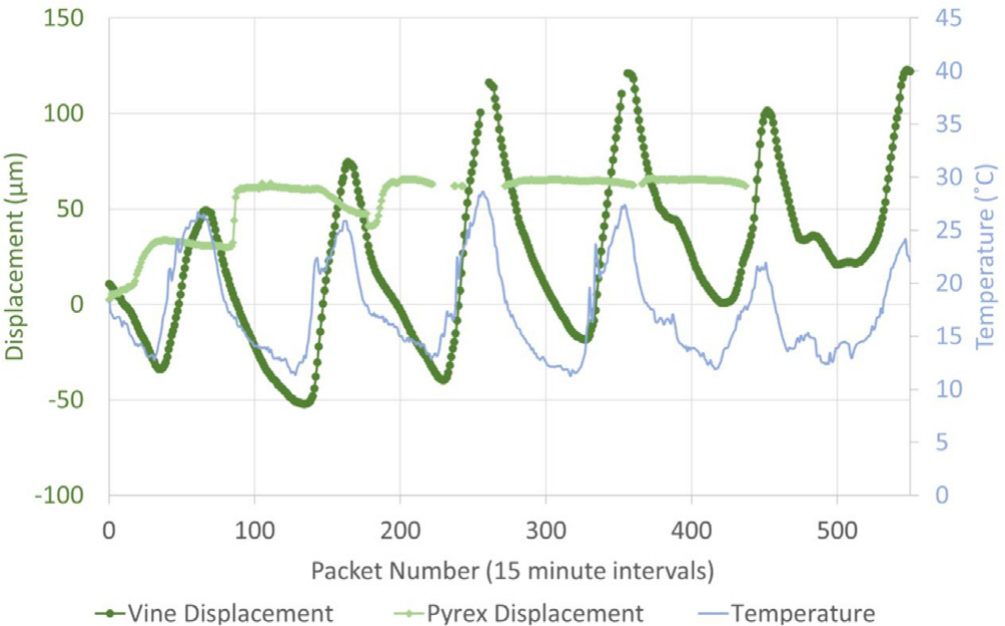
Displacement on the grapevine (dark green), displacement on a Pyrex cylinder (light green), and temperature (blue) measured by the dendrometers. Displacement is recorded as a distance from the initial position. (For interpretation of the references to color in this figure legend, the reader is referred to the web version of this article.)

Note: There are some initial jumps in displacement from the dendrometer on Pyrex; the source of these jumps has not been identified. However, the measurements seem to stabilize after about 1.5 days.

Results from Woodhall Vineyard Deployment (Fig. 58):

• Consistently tracking daily stem diameter changes of approximately 150–200 µm
• Device is mostly insensitive to temperature fluctuations
• Maximum 30 µm recorded movement per day on Pyrex cylinder

### 7.3 Vapor pressure deficit comparison

Vapor Pressure Deficit (VPD) is a function of temperature and relative humidity that measures vapor pressure in the air; it is the difference between Saturation Vapor Pressure (SVP), which is the maximum amount of water vapor that air can hold at a given temperature, and Actual Vapor Pressure (AVP), which is the true amount of water vapor in the air.

Stem diameter fluctuations are related to leaf and water potentials in plants [1], which themselves are connected to VPD. There is a strong linear correlation between VPD and leaf and stem water potential in grapevines [23]. Similar results regarding VPD and leaf / stem water potentials have been obtained from research on prune trees [24]. Plant stem diameter oscillations follow transpiration and transpiration is driven by VPD; continuous measurements of stem diameter are thereby expected to closely match VPD patterns.

**Fig. 59:**
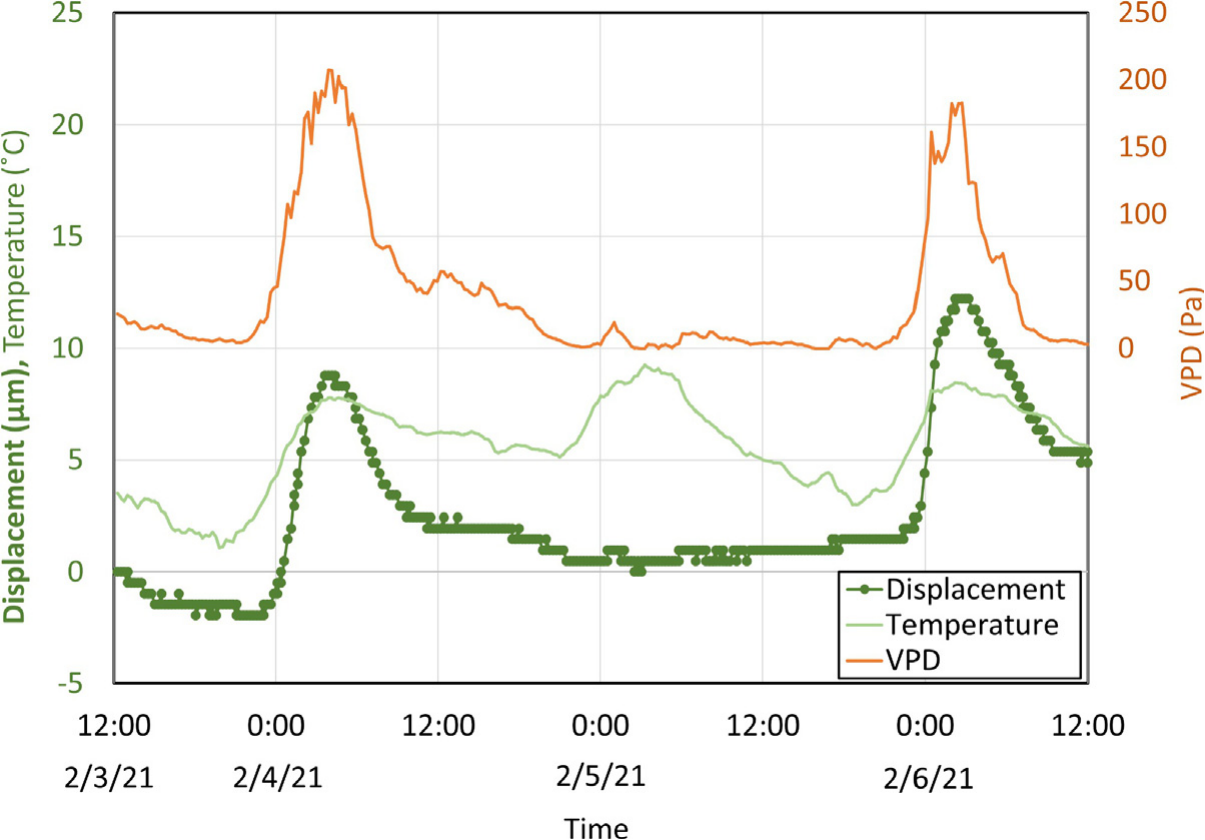
Vine displacement compared with VPD and temperature measurements from a three-day trial on a camellia plant in Corvallis, OR in February 2021. Note: Magnitude of displacement is significantly smaller than in previous trials due to early season conditions and plant species.

The OPEnS dendrometer was deployed on a camellia plant in Corvallis, Oregon for three days to compare displacement data with VPD and ambient temperature fluctuations (Fig. 59). The trends in displacement data from the dendrometer coincide with that of the VPD calculated from the temperature and relative humidity data from the SHT30 included in the dendrometer system. On the first full day (2/4/21), and third day (2/6/21), the displacement, VPD, and temperature have similar timing for their minimum and maximum values. However, despite a temperature change of 6 °C (maximum of 9 °C and minimum of 3.0 °C) on the second day of testing (Fig. 59, 2/5/21) similar to the first and third days, the displacement measurements from the dendrometer exhibit almost no change, which is consistent with the VPD trend on that day. This trial confirms that the dendrometer does not have temperature dependence and measurements accurately reflect stem diameter oscillation patterns.

Results from VPD test on camellia plant:

• Consistently tracking daily stem diameter between 15 and 33 µm
• Error tracking reported zero bugs during the three days
• Stem diameter fluctuations show a correlation with VPD and not temperature

## Device capabilities and limitations

● The Sandwich Grip can be displaced if bumped or if the plant is jostled, which could impact data acquisition and accuracy.
  ○ Best to avoid contact with a plant that has a dendrometer.
  ○ Advisable to perform a device check up (use the LED indication system) after any strong winds, plant/crop maintenance, or every couple weeks to ensure that usable data is still being collected.
● AS5311 sensor has a 0.5 µm maximum resolution [25].
  ○ Resolution of the purchased magnet must be high enough to achieve this precision (10 µm/m or better).
● Current design can accommodate vine sizes of 23–40 mm, however, mechanical components can be scaled to work on stems or vines of other sizes.
● Estimated operation time of 277 days with 3.7 V 2000mAh battery when collecting data points once every 15 min (Table 3).

**Table 3:** Dendrometer Power Budget.

Operation Mode	Current (mA)	Time (s)
Awake, measures sensors, log to SD	32	0.972
Deep sleep	0.3	900,000

Battery self-discharge: ∼5% of battery capacity per month

Battery life calculated with one battery (2000mAh):

1) $\mathrm{Battery}\;Self\;Discharge\;Rate=\frac{\mathrm{Self}\;Discharge\;Rate\;Per\;Month}{730}$
2) $\mathrm{Battery}\;Self\;Discharge\;Rate=\frac{0.05*2000}{730}=0.137mA$
3) $\mathrm{Current}\;Draw=\frac{\Sigma Current\;Draw*Time\;Period}{\Sigma TimePeriod}+Self\;Discharge\;Rate$
4) $\mathrm{Current}\;Draw=\frac{\left(32*0.972)+(0.3*900000\right)}{0.972+900000}+0.137=0.437\;mA$
5) $\mathrm{Battery}\;Life=\frac{\mathrm{Battery}\;Capacity}{\mathrm{Current}\;Draw}$
6) $\mathrm{Battery}\;Life=\frac{2000}{0.437}=4576.7\;hours=190.7\;days=6.3\;months$

## Declaration of Competing Interest

The authors declare that they have no known competing financial interests or personal relationships that could have appeared to influence the work reported in this paper.
